# An Integrative Transcriptomic and Methylation Approach for Identifying Differentially Expressed Circular RNAs Associated with DNA Methylation Change

**DOI:** 10.3390/biomedicines9060657

**Published:** 2021-06-08

**Authors:** Tianyi Xu, LiPing Wang, Peilin Jia, Xiaofeng Song, Zhongming Zhao

**Affiliations:** 1 Center for Precision Health, School of Biomedical Informatics, The University of Texas Health Science Center at Houston, Houston, TX 77030, USA; xutianyi@nuaa.edu.cn (T.X.); peilin.jia@gmail.com (P.J.); 2 Department of Biomedical Engineering, Nanjing University of Aeronautics and Astronautics, Nanjing 211106, China; 3 Department of Biobank, Clinical Medical College, Yangzhou University, Yangzhou 225001, China; NJPHWLP@163.com; 4 Human Genetics Center, School of Public Health, The University of Texas Health Science Center at Houston, Houston, TX 77030, USA; 5 MD Anderson Cancer Center UTHealth Graduate School of Biomedical Sciences, Houston, TX 77030, USA

**Keywords:** hepatocellular carcinoma, circular RNA, DNA methylation, transcriptional regulation, epigenetics

## Abstract

Recently, accumulating evidence has supported that circular RNA (circRNA) plays important roles in tumorigenesis by regulating gene expression at transcriptional and post-transcriptional levels. Expression of circRNAs can be epigenetically silenced by DNA methylation; however, the underlying regulatory mechanisms of circRNAs by DNA methylation remains largely unknown. We explored this regulation in hepatocellular carcinoma (HCC) using genome-wide DNA methylation and RNA sequencing data of the primary tumor and matched adjacent normal tissues from 20 HCC patients. Our pipeline identified 1012 upregulated and 747 downregulated circRNAs (collectively referred to as differentially expressed circRNAs, or DE circRNAs) from HCC RNA-seq data. Among them, 329 DE circRNAs covered differentially methylated sites (adjusted *p*-value < 0.05, |ΔM| > 0.5) in circRNAs’ interior and/or flanking regions. Interestingly, the corresponding parental genes of 46 upregulated and 31 downregulated circRNAs did not show significant expression change in the HCC tumor versus normal samples. Importantly, 34 of the 77 DE circRNAs (44.2%) had significant correlation with DNA methylation change in HCC (Spearman’s rank-order correlation, *p*-value < 0.05), suggesting that aberrant DNA methylation might regulate circular RNA expression in HCC. Our study revealed genome-wide differential circRNA expression in HCC. The significant correlation with DNA methylation change suggested that epigenetic regulation might act on both mRNA and circRNA expression. The specific regulation in HCC and general view in other cancer or disease requires further investigation.

## 1. Introduction

Hepatocellular carcinoma (HCC) is one of the most frequently occurring malignancies around the world [1]. Clinical investigation has shown that HCC is the sixth most common cancer and the fourth main cause of cancer mortality worldwide [2,3]. So far, early-stage HCC patients are amenable to curative therapy. The available treatment approaches for HCC include resection, liver transplantation, image-guided tumor ablation, and systemic therapy [4]. However, the prognosis of HCC patients is still not satisfactory, owing to tumor recurrence and metastasis with high frequency [5]. Therefore, there is a strong need to identify specific biomarkers for prognosis predication and new effective targets to design a powerful therapeutic approach.

During the past two decades, tremendous progress has been made in next generation sequencing technologies. Such technologies have helped investigators to reveal epigenetic abnormalities and noncoding RNA (ncRNA) change as important markers for the occurrence and progress of cancer. DNA methylation, a process through DNA methyltransferases (DNMTs) that transfers methyl groups from S-adenosyl methionine to cytosine bases of CpG dinucleotides, is one of the most studied epigenetic modifications. Previous studies have demonstrated that DNA methylation occurs predominantly at CpG sites (CpGs). Abnormal hypermethylation of the CpG sites in the promoter region of tumor suppressor genes (TSGs) could alter the chromatin spatial structure, resulting in low or no expression of TSGs [6]. DNA methylation is thus considered a promising tool for cancer diagnosis and evaluation of prognosis and treatment response [7].

Among the different types of ncRNA, circular RNA (circRNA) has become an attractive research topic due to its promising regulatory roles in cellular systems during the recent years [8,9,10,11,12,13,14]. CircRNAs represent a large class of covalently closed transcripts in multicellular organisms with emerging importance. They are derived from precursor mRNA noncanonical splicing and exhibit their roles in a tissue- and development-specific manner [14]. It has been recently reported that circRNAs can function as microRNA’s “sponges” that naturally sequester and competitively suppress microRNA (miRNA) activity [8,9]. For example, circRNA ciRS-7/CDR1as suppresses miR-7 activity, leading to an increased expression of miR-7’s target genes in brain tissue. Abnormal expression of circRNAs has been found to be associated with tumorigenesis and tumor progression [15,16]. In HCC, a previous study revealed that circTRIM33-12 is markedly downregulated in HCC tumor tissues and cell lines, and it could act as the sponge of miR-191 to suppress hepatocellular carcinoma progression, including tumor proliferation, migration, invasion, and immune evasion [17]. Most recently, circular RNAs are found to have protein-coding capacity through the special elements of internal ribosome entry site (IRES) sequence [18,19,20]. For example, Liang et al. identified a circRNA “circβ-catenin” that could encode a novel 370-amino acid β-catenin isoform. Their experimental results showed that this isoform could protect β-catenin from GSK3β-mediated degradation and potentiate the activation of Wnt/β-catenin pathway in liver cancer cells [19]. Moreover, emerging evidence indicated that some dysregulated circular RNAs contributed to DNA methylation level of downstream genes in autoimmune disease and cancer [21,22,23]. For example, Chen et al. found that a novel circular RNA, FECR1, acted as an upstream regulator to control breast cancer tumor growth by coordinating the regulation of DNA methyltransferase DNMT1 and DNA demethylase TET1 [21].

Despite accumulating evidence of circRNAs abnormally expressed in tumorigenesis, there has been little information about how circRNA expression becomes disrupted in cancer by regulation. Ferreira et al. offered some clues that circRNAs, like their corresponding linear RNAs, might undergo cancer-specific hypermethylation-associated transcriptional silencing in cancer cell lines [24]. Their results indicated that the expression of circRNAs could also be epigenetically silenced by DNA methylation. This intriguing feature between DNA methylation and circRNA has not been much explored in specific cellular conditions or diseases such as cancer. 

**Figure 1:**
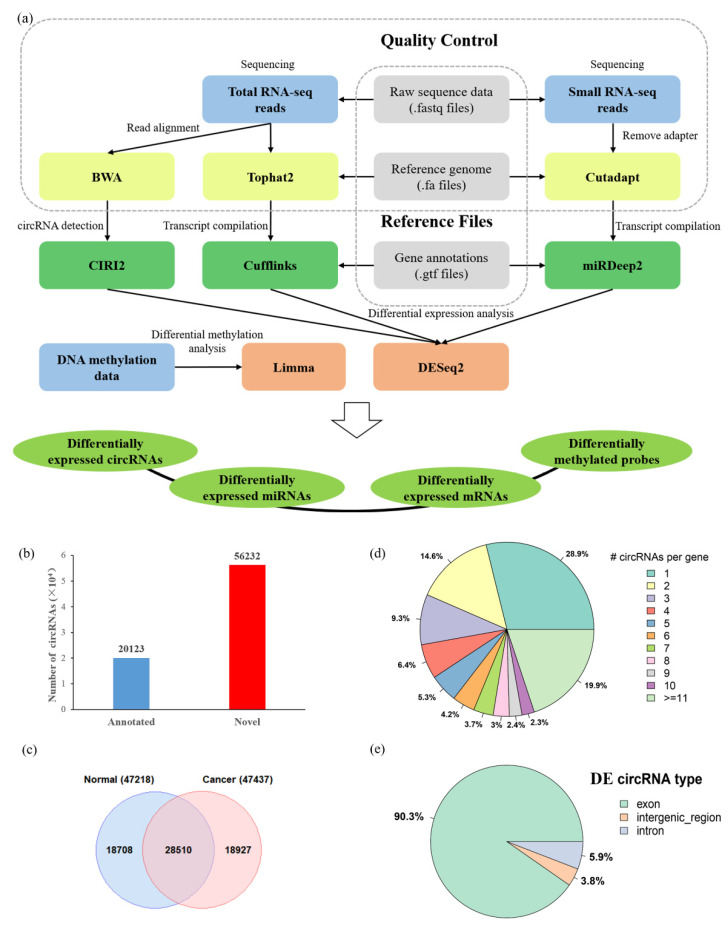
The schematic diagram of multitranscriptomic analyses for identification of differentially expressed circRNAs associated with methylation changes in hepatocellular carcinoma (HCC). (**a**) Flowchart for uncovering novel molecular signatures in HCC. (**b**) The distribution of circRNAs identified in this study according to the annotation in the circBase database or not (i.e., novel circRNAs). (**c**) Venn diagram showing the overlap of the circRNAs identified in the primary tumor versus matched adjacent normal samples. (**d**) Distribution of the number of circRNAs per gene. (**e**) The number of DE circRNAs derived from the exonic, intronic, and intergenic regions.

In this study, we explored differential circRNA profiles and their relationship with abnormal DNA methylation changes in HCC. We developed in-house computational pipelines and characterized 12,097 differentially expressed (DE) mRNAs, 312 DE miRNAs, 1759 DE circRNAs, and 191,757 differentially methylated (DM) sites based on a published dataset in HCC. Importantly, 77 circRNAs with differentially methylated sites were found to express differently in tumors versus adjacent normal samples, while their parental genes were not. Further analysis of these 77 circRNAs indicated that the expression of 34 circRNAs were significantly correlated with the alteration of DNA methylation (Spearman’s rank-order correlation, *p*-value < 0.05). We thus hypothesized that some aberrant DNA methylation events might only affect the process of pre-mRNA to generate circRNAs but not the process generating linear RNAs. In addition to testing this hypothesis, we further constructed competing endogenous RNA (ceRNA) regulation networks comprising circRNA–miRNA–mRNA pairs, from which we attempted to find potential biomarkers and possible new clues of treatments in HCC. The flowchart of our study is illustrated in Figure 1a.

## 2. Materials and Methods

### 2.1. Datasets

In this study, we retrieved several datasets from the NCBI GEO database (GSE77276) for analysis, including RNA-seq (GSE77509), DNA methylation microarray (GSE77269), and miRNA-seq (GSE76903) of primary tumors from 20 hepatocellular carcinoma patients and matched adjacent normal samples [25]. Table 1 summarizes the clinical information of the patients whose samples were used in this study.

### 2.2. Identification of Dysregulated mRNAs and circRNAs

RNA-seq data of forty samples (GSE77509) was used to identify dysregulated mRNAs and circRNAs using our pipeline. First, the raw sequence reads were cleaned by trimming the low-quality bases (Q < 20). Then, the cleaned RNA-seq reads were mapped to the human reference genome (GRCh37/hg19, UCSC Genome Browser) by Tophat2 [26] (version 2.1.1) and BWA [27] software, respectively. These two tools are capable of detecting the canonical splicing events. Next, cufflinks [26] (version 2.2.1) was used to assemble mRNAs, and CIRI2 (version 2.0.6) [28,29] was used to identify circRNAs by recognizing the back-splicing junction (BSJ) reads. CIRI2 is a program designed to differentiate BSJ reads from non-BSJ reads using efficient maximum likelihood estimation (MLE) based on multiple seed matching and to filter false positives derived from repetitive sequences or the mapping errors. To evaluate the relative expression of mRNAs and circRNAs between tumor versus adjacent normal samples, DESeq2 [30], an R package for differential expression analysis based on negative binomial generalized linear models, was used with the cutoff values (adjusted *p*-value < 0.05, |log2 (fold change)| > 1 for mRNA, adjusted *p*-value < 0.05 and junction read counts ≥ 2 for circRNAs). The read counts mapped to the sequence of mRNAs and mapped to the BSJ of circRNAs were input to DESeq2 for differential expression analysis, respectively. 

### 2.3. Small RNA Data Analysis

Mature miRNA and precursor miRNAs of human were obtained from miRBase [31]. The raw reads of 40 miRNA-seq samples (GSE76903) were first subjected to adapter removal through the Cutadapt program (version 1.9) [32]. Then, the clean reads were further preprocessed by miRDeep2 [33]. The known mature miRNA expression profile was generated by using the quantifier module of the miRDeep2 package that gives the read counts for the known miRNAs. The differentially expressed miRNAs were determined by DESeq2 with the cutoff of adjusted *p*-value < 0.05 and |log2 (fold change)| > 1.

### 2.4. Differential Methylation Analysis

The definition of the “promoter” region in a circRNA is currently an unsolved issue. In this study, for methylation analysis, we extracted three regions of a circRNA: a 2 kb sequence immediately upstream of the back-splicing site (Pre2000), the circRNA’s sequence (Interior), and a 2 kb sequence immediately downstream of the back-splicing site (After2000). For each gene, its promoter, defined as the 2 kb sequence immediately upstream of the TSS, and gene body region were selected for methylation analysis.

The relative methylation levels were measured as β-values ranging from 0 to 1, where a value close to 0 indicates low level of DNA methylation, and a value close to 1 indicates high level of DNA methylation. Because M-values are statistically valid for differential methylation analysis [34], we converted the original β-values to M-values through logistic transformation. Based on the M-values, the R package limma [35] was used to identify the differentially methylated sites between tumor and adjacent normal samples. The limma method uses the linear models and empirical Bayes methods, which can produce stable analyses from experiments with a small sample size, to assess the difference between the two groups. Because stringent multiple testing may produce a high false-negative rate when the number of samples is small, we used adjusted *p*-value < 0.05 and M-value difference (|ΔM|) > 0.5 as the cutoffs to identify the significantly differentially methylated sites.

### 2.5. Prediction of miRNAs Related to circRNAs

To predict the relationship between dysregulated miRNAs and differentially expressed circRNAs with differentially methylated sites, the sequences and annotations of miRNAs were obtained from the miRBase database. Then, miRanda [36] (August 2010 Release) and TargetScan [37] (Release 7.1) pipelines were used to predict the circRNA–miRNA interaction network. In the miRanda pipeline, we set parameters with the match score higher than 140 and the minimum free energy less than −20 to improve the reliability of our prediction.

### 2.6. Construction of the circRNA–miRNA–mRNA Network

To predict the potential roles of DE circRNAs with DM sites in molecular regulation, the circRNA–miRNA–mRNA networks were constructed by combining the circRNA–miRNA and miRNA–mRNA pairs. We used the experimentally validated miRNA–target interaction datasets from miRTarBase [38] (Release 7.0) to filter the DE miRNAs–DE mRNA pairs in our study. Then, network visualization software Cytoscape (version 3.7.1) [39] was used to display the circRNA–miRNA–mRNA regulation network in HCC. 

### 2.7. Integrated Functional Enrichment Analysis

The corresponding parental genes of circRNAs and the significantly dysregulated mRNAs in ceRNA network were both used for the functional enrichment analysis. Gene Ontology (GO) enrichment analysis (including its three domains: biological processes, cellular components and molecular function) and Kyoto Encyclopedia of Genes and Genomes (KEGG) pathways were performed using WEB-based Gene Set Analysis Toolkit (WebGestalt) [40] with default parameters and false discovery rate (FDR) < 0.05.

### 2.8. Survival Analysis of Target Genes

We performed survival analysis and drew the survival curves using an online database, GEPIA2 [41], and UALCAN [42]. These tools provide the relationship between patient survival information and gene expression based on The Cancer Genome Atlas (TCGA) datasets. We chose the liver hepatocellular carcinoma (LIHC) and input the target genes in the ceRNA predicted network to explore the survival curves. The genes with *p*-value < 0.1 were considered critical candidate genes.

### 2.9. Conventional Classification Algorithms

Six conventional machine learning classifiers, including Decision Tree (DT), Gradient Boosting (GB), K-Nearest Neighbor (KNN), Logistic Regression (LR), Random Forest (RF) and Support Vector Machine (SVM), were implemented. Each classification algorithm was trained using the expression feature of DE circRNAs in HCC. The receiver operating characteristic curve (ROC) was drawn, and then the area under the curve (AUC) value was calculated based on the 10-fold cross-validation (CV) to evaluate each algorithm’s performance. 

Table S1 provides the list of bioinformatics tools used in the analyses.

## 3. Results

### 3.1. The Landscape of Differentially Expressed circRNAs in HCC

Our genome-wide transcriptomic analysis identified over 10,000 circRNAs in each of the 20 paired matched tumor and adjacent normal samples with at least two junction supporting reads by CIRI2 (Figure 1a). To improve the analysis confidence of our study, we focused on the circRNA that could be identified in at least two samples, resulting in 76,355 unique circRNAs that were detected. After comparing with the previously published circRNAs deposited in database circBase [43] (containing 140,790 human circRNAs), we found that the overlap of circRNAs in our study and circBase was significant (Fisher’s Exact Test, *p*-value < 2.2 × 10^−16^): 20,123 circRNAs have been annotated in the circBase, while a total of 56,232 were novel circRNAs (Figure 1b). Furthermore, 47,437 and 47,218 circRNAs were respectively found in tumor and normal groups. Among them, 28,510 circRNAs were present in both groups, indicating the overlap of circRNAs in tumor and normal samples was not significant (Figure 1c, Fisher’s Exact Test, *p*-value > 0.05). Notably, we found that 28.9% (2968/10,280) of circRNA-producing genes generated only one circRNA, and 19.9% (2046/10,280) genes produced more than 10 circRNAs (Figure 1d). Of note, the gene *DNAH14* generated 127 circRNAs, and the gene *BIRC6* generated 124 circRNAs. This is likely because each of them has large number of exons (*DNAH14* and *BIRC6* have 90 and 78 exons, respectively) in the human genome.

**Figure 2:**
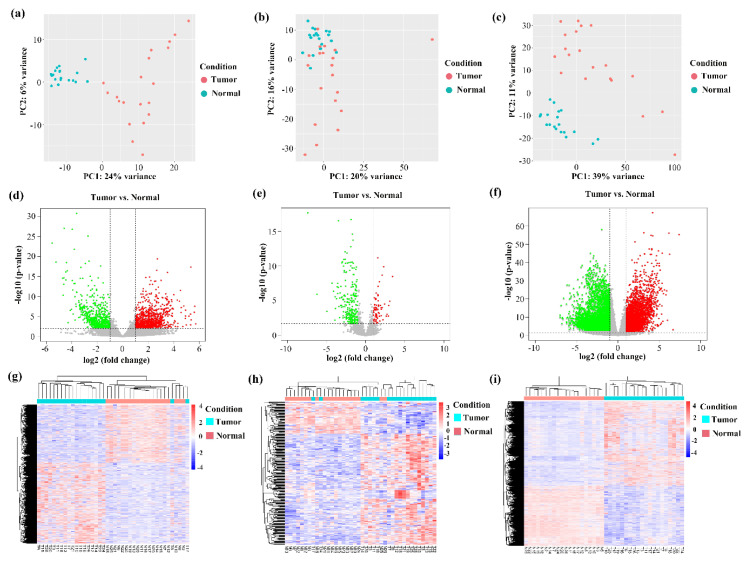
Analysis of circular RNA (circRNA), microRNA (miRNA), and mRNA expression in hepatocellular carcinoma (HCC). (**a**–**c**) Principal component analysis of circRNA (**a**), miRNA (**b**), and mRNA (**c**) expression in primary tumor and adjacent normal samples, respectively. Each dot represents one sample. (**d**–**f**) Volcano plots showing differentially expressed circRNAs (**d**), miRNAs (**e**) and mRNAs, (**f**) in HCC, respectively. Red and green dots denote significantly upregulated and downregulated RNAs, respectively. (**g**–**i**) Heatmap displaying the significantly dysregulated circRNAs (**g**), miRNAs (**h**), and mRNAs (**i**) in HCC, respectively. X-axis: sample IDs (tumor samples: starting with a T; normal samples: starting with a N).

**Figure 3:**
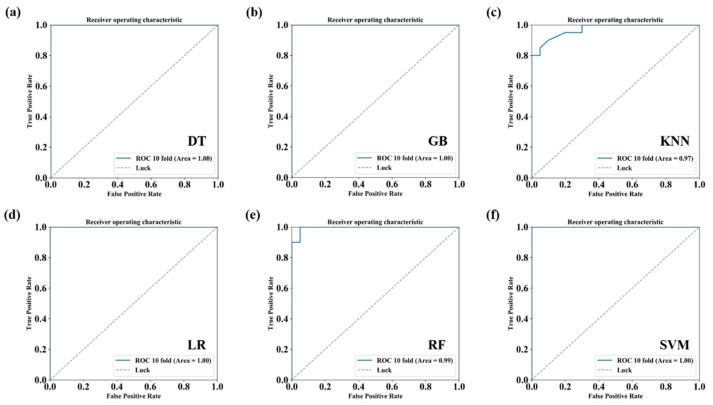
Performance of six machine learning methods (DT, GB, KNN, LR, RF, and SVM, as shown in Figure 3a–f) on differentially expressed circRNAs for predicting hepatocellular carcinoma. DT: Decision Tree. GB: Gradient Boosting. KNN: K-Nearest Neighbor. LR: Logistic Regression. RF: Random Forest. SVM: Support Vector Machine.

Next, we used DESeq2 to analyze the differentially expressed circRNAs (DE circRNAs) between tumor and adjacent normal samples. The results showed that 1759 circRNAs were differentially expressed (adjusted *p*-value < 0.05). The top 20 differential expressed circRNAs in HCC are shown in Table 2. According to the chromosome location of circRNAs, we concluded that more than 90% of DE circRNAs were back-spliced from exonic regions; however, we also found 6% DE circRNAs and 4% DE circRNAs derived from intergenic regions and intronic regions, respectively (Figure 1e). Principal component analysis (PCA) showed that circRNA expression could distinguish tumor and adjacent normal samples well (Figure 2a). Furthermore, we applied six conventional machine learning algorithms (DT, GB, KNN, LR, RF, and SVM) to conduct prediction models based on the expression of DE circRNAs. As shown in Figure 3, each model has a high AUC value (AUC = 0.97~1.00). The results suggested that circRNAs might have the potential roles to serve as biomarkers for predicting HCC (Figure 3). Further statistical analysis in HCC tumor sample versus the adjacent normal sample revealed that 1012 circRNAs (57.53%) were upregulated, while 747 circRNAs (42.47%) were downregulated in HCC (Figure 2d). Figure 2g displays the heatmap cluster of DE circRNAs across 40 samples. The results showed that tumor and adjacent normal samples could be categorized into two different branches, except for T8 and T17 samples.

We performed gene set enrichment analysis of the parental genes of dysregulated circRNAs using GO terms and KEGG pathways and online tool WebGestalt (version 2019). The GO enrichment results indicated that these parental genes had enrichment function in cellular response to stress (GO: 0033554, biological process, *p*-value = 2.45 × 10^−8^), nucleoplasm part (GO: 0044451, cellular components, *p*-value = 5.77 × 10^−15^), and adenyl ribonucleotide binding (GO: 0032559, molecular function, *p*-value = 4.18 × 10^−11^). Nine significant pathways were also found by KEGG pathways analysis (FDR < 0.05, Figure S1). Among them, the pathway of synthesis and degradation of ketone bodies (hsa00072) and the valine, leucine, and isoleucine degradation pathway (hsa00280) were previously reported to be associated with liver metabolic function and play important roles in the progression of HCC [44,45].

### 3.2. The Differential Expression Pattern of circRNAs and Genes in HCC

Genome-scale gene expression of primary tumor and matched adjacent normal samples for 20 HCC patients were examined by the tools in Figure 1a. Principal component analysis of the genes revealed a significant differentiation between the tumor and normal samples (Figure 2c).

DESeq2 was used to identify the DE genes based on read counts. By statistical analysis, we drew volcano plots for the genes in HCC paired samples (|log2 (fold change)| > 1, adjusted *p*-value < 0.05) (Figure 2f). In total, 12,097 differentially expressed genes were identified. Among them, 7087 genes (58.58%) were upregulated, and 5010 genes (41.42%) were downregulated in tumor samples. As shown in Figure 2i, all HCC samples were clustered together by gene expression data. After combining the DE circRNAs’ expression in Figure 2g, the results suggested that HCC samples had distinct transcriptomic changes at both the gene and circRNA molecular levels when compared to control samples. 

**Figure 4:**
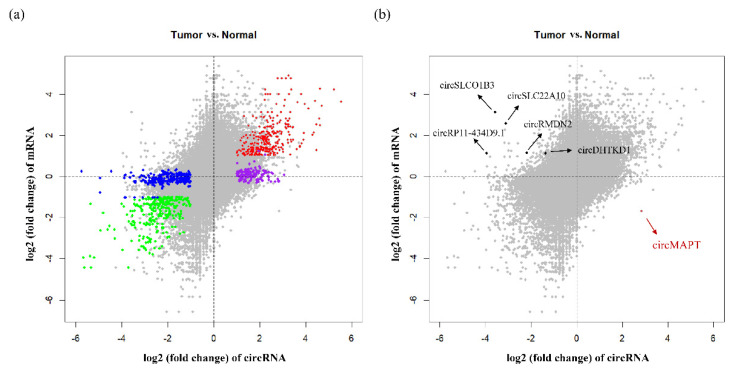
The correlation between log2 (fold change) of circRNAs and log2 (fold change) of linear mRNAs in HCC. (**a**) Red and green data points denote the differential expression of the circRNAs that were positively correlated with the differential expression of the linear mRNAs. Blue and purple data points denote the circRNAs that were differentially expressed in tumor samples with no corresponding change in parental gene expression. (**b**) Bold black and brown data points denote the circRNAs whose differential expression had negative correlation with the differential expression of the linear mRNAs.

Because circRNAs were derived from their precursor mRNA (pre-mRNA) splicing process, we examined the expression level of DE circRNA’s parental genes and compared the expression pattern of circRNAs and their parental genes in HCC. Interestingly, despite that the dysregulation of majority DE circRNAs could be explained by the dysregulation of the parental genes (Figure 4a, red and green data points), we also found 371 DE circRNAs whose parental genes showed no obvious change in HCC (Figure 4a, blue and purple data points). Further analysis showed that 187 parental genes corresponding to upregulated circRNAs and 147 parental genes corresponding to downregulated circRNAs were not significantly differentially expressed, respectively. More importantly, we characterized that the expression patterns of six circRNAs were opposite to their parental genes (Figure 4b, black and brown data points), confirming previous findings that the expression of circRNAs can be independent of their parental genes [46]. We further checked their parental genes’ expression using the data from the Genotype-Tissue Expression (GTEx, https://gtexportal.org/home/, accessed on 15 October 2019) project. Interestingly, we found four genes (*RP11-434D9.1*, *DHTKD1*, *SLC22A10*, and *SLCO1B3*) had the highest expression level in liver among all the GTEx tissues, while one gene (*MAPT*) showed no expression in liver (below the cutoff value). The opposite expression pattern between circRNA and parental gene suggested that circRNAs could serve as valuable biomarkers in hepatocellular carcinoma.

We performed enrichment analysis for the parental genes that showed no significant expression change, but the corresponding circRNAs were significantly differentially expressed. A total of nine GO terms were significantly enriched in 234 genes (Figure S2a), including biological process terms such as regulation of insulin receptor signaling pathway (GO:0046626, *p*-value = 5.56 × 10^−6^), regulation of cellular response to insulin stimulus (GO:1900076, *p*-value = 9.02 × 10^−6^), cellular component terms such as nucleoplasm part (GO:0044451, *p*-value = 4.98 × 10^−6^), nuclear body (GO:0016604, *p*-value = 9.07 × 10^−6^), and molecular function terms such as GTPase binding (GO:0051020, *p*-value = 1.67 × 10^−5^). In addition, the enrichment analysis using KEGG pathways revealed that these genes were primarily enriched in ubiquitin mediated proteolysis (*p*-value = 4.21 × 10^−5^, Figure S2b). Previous studies have reported that this pathway was known to be important for hepatocarcinogenesis [47,48,49]. To better interpret the results, we constructed a network for those enriched GO terms (Figure S2c). From this network, we found some genes associated with multiple terms. For example, *NR1H4* was involved in three terms. *NR1H4* regulated bile acid synthesis, transport, and catabolism. It encoded a core transcription factor for normal liver homeostasis [50,51]. Taken together, these findings indicated that these special DE circRNAs may have potential regulatory function in HCC progression.

### 3.3. Altered DNA Methylation Events Associated with circRNAs in HCC

The DNA methylation datasets of 20 HCC patients were generated by the Illumina Infinium HumanMethylation450K BeadChip Kit, which assays for more than 485,000 CpG sites. Similar to gene methylation analysis, three regions (Pre2000, Interior, and After2000) were selected to investigate the methylation patterns in circRNAs. Specific details are described in Materials and Methods.

By combining the circRNA expression with the analysis results of genome-scale DNA methylation, we drew volcano plots to display differentially expressed circRNAs that also harbored the DM sites (Figure S3, blue and black data points). Our analysis found that 195 upregulated (195/1012, 19.27%) and 134 downregulated (134/747, 17.94%) DE circRNAs had DM sites in HCC (adjusted *p*-value < 0.05 and |ΔM| > 0.5). Furthermore, we investigated the distribution of DE circRNAs with or without harboring DM site across human chromosomes. The number of circRNAs was normalized by gene density in each chromosome. The results indicated that chromosome 10 had the highest proportion of DE circRNAs, while chromosome 19 had the lowest proportion in HCC. Of note, chromosome 19 had the highest density of gene among all the human chromosomes [52]. When we compared the number of DE circRNAs with that of DM sites, we found that chromosome 13 had the highest ratio, while chromosome 20 had the lowest ratio, after excluding the sex chromosomes (Figure S4).

Next, in the upregulated DE circRNA group, 28 (23 hypermethylated and 5 hypomethylated), 196 (179 hypermethylated and 17 hypomethylated), and 30 (27 hypermethylated and 3 hypomethylated) significant DM sites were detected, respectively, in After2000, Interior, and Pre2000 regions of circRNAs. In downregulated DE circRNA group, 27 (19 hypermethylated and 8 hypomethylated), 143 (113 hypermethylated and 30 hypomethylated), and 23 (20 hypermethylated and 3 hypomethylated) significant DM sites were detected, respectively, in After2000, Interior, and Pre2000 regions of circRNAs (Table 3). These results revealed that the number of hypermethylated sites was higher than that of hypomethylated sites in DE circRNAs, and both were mainly distributed in interior regions (>80%). 

**Figure 5:**
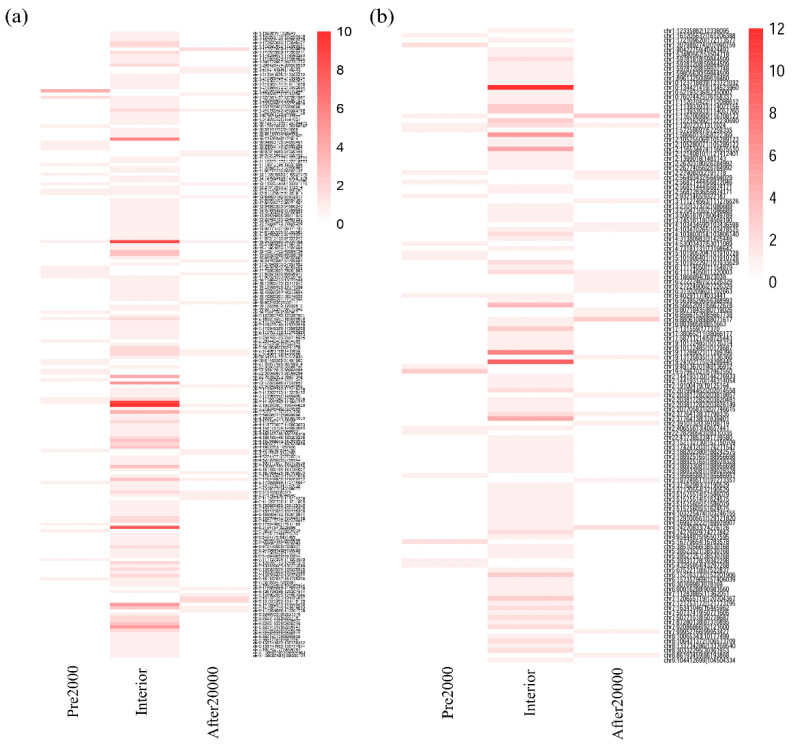
Heatmap showing the number of differentially methylated sites in differentially expressed upregulated circRNAs (**a**) and downregulated circRNAs (**b**).

We further analyzed and displayed the DM site frequency for each DE circRNA by heatmap (Figure 5). We found 88.7% (173/195) upregulated circRNAs and 92.5% (124/134) downregulated circRNAs had only one region with DM sites. Interestingly, circSNHG14 (chr15:25328543-25339104) and circTOLLIP (chr11:1307232-1317024) had DM sites in all regions in both the upregulated and downregulated groups. On the other hand, most DM sites distributed in the circRNA interior regions (79.5% upregulated and 76.1% downregulated). Moreover, chr3:195343807-195444620 and circINPP5A (chr10:134421419-134523960) had more than 10 DM sites in the interior regions.

### 3.4. Aberrant DNA Methylation Profiles May have Specific Effect on Circular RNAs That Are Not Observed on Linear RNAs in HCC

We analyzed the methylation level of these DE parental genes corresponding to DE circRNAs. The results showed that 69 DE parental genes of upregulated circRNAs and 49 DE parental genes of downregulated circRNAs did not have DM sites (Table 4).

By combining the circRNA expression, mRNA expression, and DNA methylation changes in our study, we found a total of 77 DE circRNAs (46 upregulated and 31 downregulated) had significant DM sites, while their parental genes had no differential expression in tumor versus normal samples. Further analysis showed that the expression of 34 DE circRNAs (18 upregulated and 16 downregulated) had significant correlation with alteration of DNA methylation in HCC, and 9 circRNAs were significantly correlated with more than one DM site (Spearman’s rank-order correlation, *p*-value < 0.05, Table S2). 

By further examining the sequence component of these DE circRNAs harboring DM sites, we found that most of them (>90%) contained more than three exons. Notably, our analysis revealed that alternative splicing events of circAHSA2P (chr2:61406116-61413889), circDCUN1D4 (chr4:52729603-52765544), and circCLEC16A (chr16:11114050-11220003) were different from their corresponding parental mRNAs. That is, the circ–exon sequences are derived from different linear transcripts. These results further support that the process of generating circRNAs and mRNAs may be different. Strong evidence has been implicated that the circCLEC16A’s parental gene *CLEC16A* plays important role in autoimmunity, and the variation in *CLEC16A* was associated with multiple immune-mediated diseases, such as type 1 diabetes, multiple sclerosis, and systemic lupus erythematosus [53,54,55,56,57]. Thus, we considered that *CLEC16A* may act through generating circRNA to perform biological function in HCC. GO analysis showed no significant enrichment of 77 circRNAs’ parental genes (FDR > 0.05); however, we noticed that circCCNL2 (chr1:1322615-1326245) was derived from cyclin L2 gene (*CCNL2*). A previous study has demonstrated that *CCNL2* was involved in pre-mRNA splicing and induced apoptosis of human hepatocellular carcinoma cells [58]. Although RNA-seq analysis showed that the expression of *CCNL2* did not change at mRNA level when compared to controls, the dysregulated circRNAs observed from *CCNL2* gene in HCC likely represent a novel mechanism underlying apoptosis in human hepatocellular carcinoma cells. We hypothesized that the alteration of DNA methylation may influence the process of pre-mRNA splicing and such a process prefers to influence the transcription factors (TFs) that mediate circRNAs’ generation rather than the TFs mediate linear mRNAs’ generation. According to this hypothesis, aberrant DNA methylation may have specific regulation on circular RNA. Such a role may not similarly act on linear RNA.

### 3.5. Construction of ceRNA Regulatory Network in HCC

**Figure 6:**
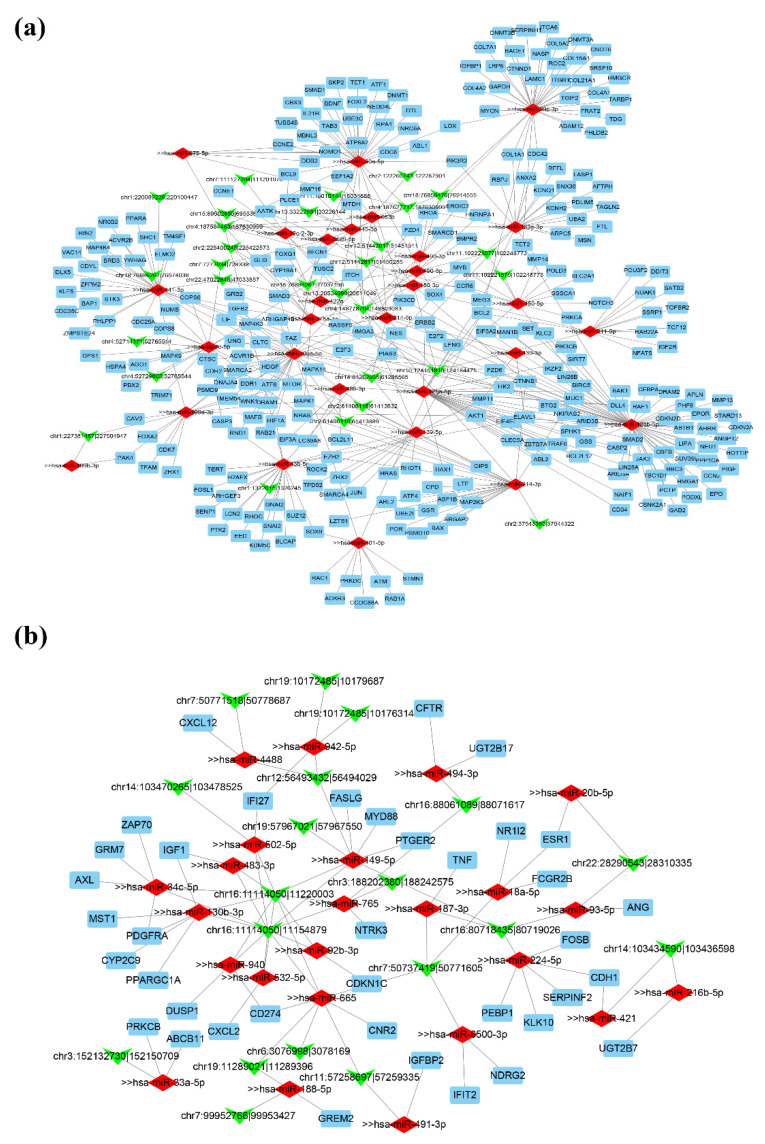
Competing endogenous RNA (ceRNA) regulatory network. (**a**) The network consists of 29 upregulated circRNAs harboring differentially methylated sites, 29 downregulated microRNAs, and 299 upregulated mRNAs. (**b**) The network consists of 19 downregulated circRNAs harboring differentially methylated sites, 22 upregulated microRNAs, and 38 downregulated mRNAs. Green, red, and blue nodes represent circRNAs, microRNAs, and mRNAs, respectively. Edge denotes their relationship.

Regulatory network is an important tool to study cancer such as HCC [59,60], as previous studies have reported that circRNAs could compete with miRNAs and regulate miRNA-mediated target genes. In this study, we constructed ceRNA regulation network to further explore the potential function and regulatory mechanisms of circRNAs harboring DM sites while we excluded those parental genes with differential expression in HCC. First, we used DESeq2 to identify the differentially expressed miRNAs in HCC. In total, 312 DE miRNAs were identified (|log2 (fold change)| > 1, adjusted *p*-value < 0.05, Figure 2e). Among them, 243 miRNAs (77.88%) were upregulated, while 69 miRNAs (22.12%) were downregulated in tumor tissues (Figure 2h). Then, we used miRanda and TargetScan pipelines to test which DE miRNAs could interact with these 77 DE circRNAs (see details in Materials and Methods). Next, we used the experimentally validated miRNA-target interaction datasets from miRTarBase [38] (Release 7.0) and the DE mRNAs in our study to predict the potential role for these special DE circRNAs in molecular regulation. Finally, Cytoscape software was used to visualize the circRNA–miRNA–mRNA regulation network. Taken together, two genome-wide networks were constructed according to the relationships between co-expressed circRNAs and miRNAs and between miRNAs and mRNAs: Figure 6a shows 29 upregulated circRNAs, 29 downregulated miRNAs, and 299 upregulated mRNAs. Figure 6b displays 19 downregulated circRNAs, 24 upregulated miRNAs, and 38 downregulated mRNAs. Among them, circCLEC16A_1 (chr16:11114050-11154879) and circCLEC16A_2 (chr16:11114050-11220003) (downregulated in HCC samples) interacted with the largest number (*n* = 10) of upregulated miRNAs, suggesting these two circRNAs may be important regulatory factors in HCC. 

### 3.6. Survival Analysis of Target Genes in Network

**Figure 7:**
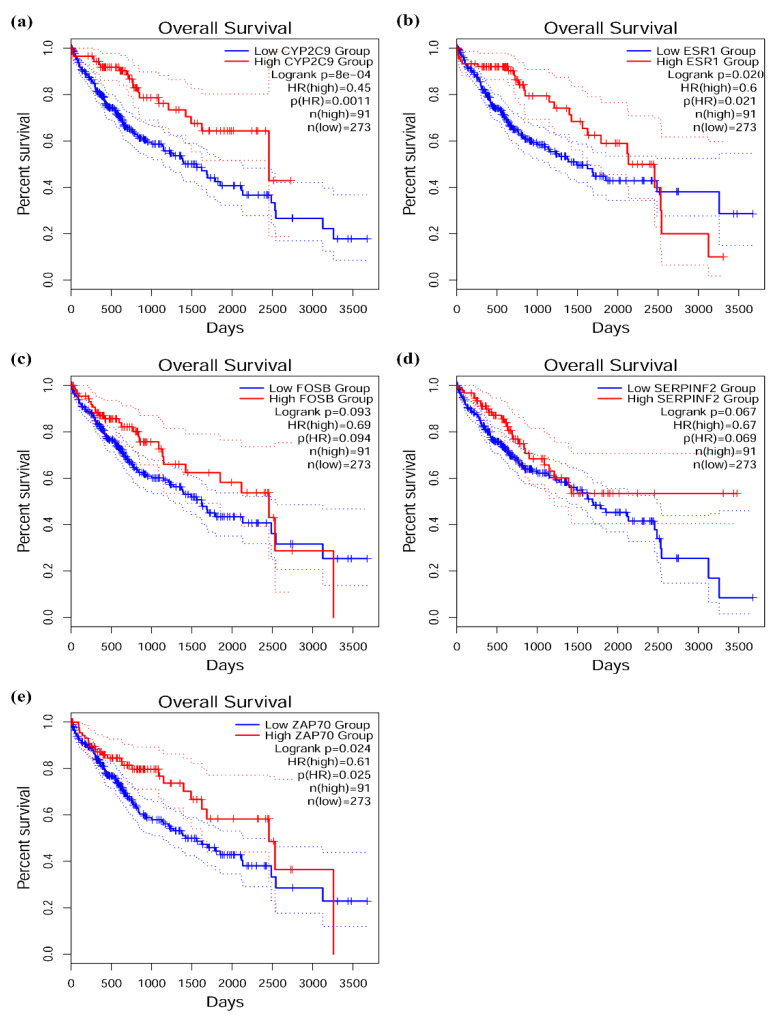
Target genes with significant correlation with survival time in downregulated competing endogenous RNA (ceRNA) network. (**a**) *CYP2C9*. (**b**) *ESR1*. (**c**) *FOSB*. (**d**) *SERPINF2*. (**e**) *ZAP70*. Statistical significance: *p*-value < 0.1.

To explore the relationship between our identified target genes and clinical features, we performed survival analysis and drew the survival curves using an online database, GEPIA2 [41], and UALCAN [42]. These tools provide the relationship between patient survival information and gene expression base on The Cancer Genome Atlas (TCGA) datasets. We chose the liver hepatocellular carcinoma (LIHC) and input the target genes in the ceRNA predicted network to explore the survival curves. In upregulated circRNA network, we found that 137 target genes (45.8%) were significantly correlated with survival in HCC. Notably, a high expression level of them revealed a significantly poor overall survival (OS). (Figure S5). Moreover, in downregulated circRNA network, five target genes (13.2%) were significantly correlated with survival outcome in HCC. Interestingly, a low expression level of these genes (*CYP2C9*, *ESR1*, *FOSB*, *SERPINF2*, and *ZAP70*) was associated with a significantly poor OS. (Figure 7).

To explore the potential molecular therapeutic targets for HCC, we decomposed the ceRNA regulatory network. *CYP2C9* could be inhibited by miR-130b-3p, while two circRNAs, circCLEC16A_1 and circCLEC16A_2, may act as “sponges” to mediate the expression of miR-130b-3p. In addition, based on the network, circCLEC16A_2 may regulate *ZAP70* through miR-34c-5p, and circCDYL2 may regulate the expression of *FOSB* and *SERPINF2* through miR-224-5p. CircGRB10 and circTTC28 interacted with miR-18a-5p and miR-20b-5p, respectively, to regulate the expression of gene *ESR1*, which was detected as a candidate tumor suppressor gene and reported to be associated with the susceptibility to persistent HBV infection [61,62]. Collectively, these DE circRNAs associated with DNA methylation changes in our regulatory network are promising candidates for future study in HCC. Further studies will be warranted for validation some of these circRNAs.

## 4. Discussion

HCC accounts for >80% of primary liver cancers and is also the fourth main cause of cancer mortality worldwide [3]. Although the available treatment approaches for early-stage HCC include resection, liver transplantation has been developed [4]. The prognosis of HCC patients is still not satisfactory, owing to tumor recurrence and metastasis with high frequency [5]. Therefore, further studies are needed to identify specific biomarkers for prognosis predication and new effective targets to design a powerful therapeutic approach. In recent years, numerous studies have reported that circRNAs possess important functions in cancer [15,16], and their expression, like the linear RNAs, can be epigenetically silenced by DNA methylation [24]. However, circRNA expression and its correlation with DNA methylation have not been well profiled in cancer, including HCC. To better understand the molecular mechanisms underlying HCC development and progression, we systematically analyzed the expression changes at multitranscriptional levels and regulatory networks, including differentially expressed mRNAs, miRNAs, circRNAs, and DNA methylation alternation through high-throughput RNA sequencing. Our study represents the first comprehensive characterization of the potential relationship between DNA methylation and circRNAs in HCC. 

In our work, we characterized a total of 12,097 differentially expressed mRNAs, 312 DE miRNAs, 1759 DE circRNAs, and 191,757 differentially methylated sites. According to PCA analysis, HCC tumor and matched normal samples were better distinguished by circRNA and mRNA expression than by miRNA expression (Figure 2g–i). This observation implied that circRNAs could be potential biomarkers for diagnose and prognosis of HCC in future. In our DE analysis, we found that some circRNAs were significantly differentially expressed, but the corresponding parental genes showed no significant expression change in HCC. This unique gene set is particularly interesting to study the potential roles of DE circRNAs in the disease like HCC. The GO function and KEGG pathway analysis revealed many enriched gene sets in liver function or hepatocarcinogenesis, indicating that circRNAs may have potential regulatory function in HCC progression. Furthermore, we observed that the expression pattern of six circRNAs were opposite to their parental genes. In our examination of expression of tissue panel using the GTEx data, we found that four genes (*RP11-434D9.1*, *DHTKD1*, *SLC22A10*, and *SLCO1B3*) had highest expression level in liver than any other tissues in the human body. The opposite expression pattern suggested that circRNAs combined with mRNAs could serve as paired biomarkers in HCC. 

So far, we have known that some circRNA expression changes are associated with cancer progression, but the knowledge of circRNA silenced by DNA methylation in HCC has been very limited. Thus, our study had a unique way to perform epigenetic analysis. We detected 195 upregulated and 134 downregulated DE circRNAs that had DM sites in After2000, Interior, or Pre2000 regions of the circRNAs. Most of the DM sites (84.3%) were hypermethylated (Table 3) and mainly distributed in their interior regions (>80%, Figure 5). 

A key finding of our study was that 46 upregulated and 31 downregulated circRNAs had significant DM sites, but their parental gene had no differential expression in HCC. This specific set of circRNAs may be directly regulated by DNA methylation to link to the potential clinical outcome. For example, circSLC43A1 (chr11:57258697-57259335) was downregulated in tumor versus normal samples. The expression level of circSLC43A1 was significantly positively correlated with altered methylation level of cg11376147 probe in HCC (Spearman’s rank-order correlation, *p*-value < 0.05), while the parental gene *SLC43A1* was not differentially expressed. Considering circRNAs and mRNAs were both generated from pre-mRNA through different splicing methods, this finding indicated that some aberrant DNA methylation events might have specific effect on circular RNA but not linear RNA in HCC. Nano et al. reported that cg11376147 probe annotated to *SLC43A1* gene was associated with gamma–glutamyl transferase (GGT) level in whole blood [63]. GGT is an enzyme elevated in the blood in most diseases, causing damage to the liver. Our results provided new insights into epigenetic mechanisms that circRNAs regulated by DNA methylation could be potential markers associated with liver function or liver cancer. 

Regulatory networks have been established to understand DE circRNA regulation pattern in our study. The networks contained DE circRNAs harboring differentially methylated sites, DE miRNAs, and DE mRNAs in HCC. The network analysis identified many potential regulator modules that might play important roles in HCC. Among them, circCLEC16A_1 and circCLEC16A_2 (downregulated in HCC samples) interacted with the largest number (*n* = 10) of upregulated miRNAs in network, suggesting these two circRNAs may be important regulatory factors in HCC. Furthermore, our analysis of TCGA dataset found that a total of 142 target genes in the regulated network were correlated with survival curves with clinical outcome significance in HCC. We expect it will provide potential candidates for future functional studies of circRNAs in HCC.

This study has some limitations or potential future work. First, genome-wide analysis may lead to both false positive and false negative results. Experimental validation using matched tissues (e.g., HCC and adjacent tissues) will be needed in future study. Second, circRNA and other gene annotations are still limited. Third, we may expand our work by using some additional public data. For example, The Cancer Genome Atlas (TCGA) has RNA and miRNA sequencing data, along with the methylation and clinical data. Although the number of samples with matched total RNA-seq, small RNA-seq, and methylation data is expected to be small in each cancer type, this unique resource may help us further explore such synergistic features in pan-cancer. Such data may also allow us to perform survival analysis to validate the selected circRNAs as potential biomarkers in cancer.

## 5. Conclusions

In summary, we performed a unique integrative omics analysis of circRNAs with differential gene expression and methylation change. We reported a list of circRNAs whose expression was associated with DNA methylation alteration, but their parental genes were not. This list of circRNAs may serve as promising candidates to further explore their roles in gene regulation, especially how such regulation is coordinated with the protein-coding mRNA expression regulation.

## Figures and Tables

**Table 1:** The clinicopathological characteristics of patients used in this study.

Patient ID	Gender	Age (Years)	Tumor Size (cm)	Hbv Infection
#3	female	46	6	Yes
#6	male	35	14.1	Yes
#7	male	42	38	Yes
#8	male	61	8	No
#10	male	66	12	Yes
#11	male	53	8	Yes
#12	female	49	10	Yes
#13	male	52	17	Yes
#14	female	51	5.5	Yes
#15	male	47	5	Yes
#16	male	43	10	Yes
#17	male	60	3	Yes
#18	male	61	10	Yes
#19	male	43	7	Yes
#20	male	64	10	Yes
#21	male	40	7	NA
#22	male	53	19	Yes
#24	male	62	2.4	Yes
#25	male	48	6.7	Yes
#26	male	49	NA	Yes

**Table 2:** Top 20 differentially expressed circRNAs in HCC.

circRNA ID	circRNA Type	Gene ID	Gene Symbol	log2 (Fold Change)	p_adj
chr12:96381971-96384310	exon	ENSG00000084110.6	*HAL*	−3.63	1.96 × 10^−^^27^
chr10:96701615-96732002	exon	ENSG00000138109.9	*CYP2C9*	−4.61	5.57 × 10^−^^24^
chr19:10183600-10184111	exon	ENSG00000167798.12	*C3P1*	−3.97	6.33 × 10^−^^24^
chr3:51575514-51586079	intron	ENSG00000164080.9	*RAD54L2*	−2.73	2.43 × 10^−^^22^
chr16:56385296-56388993	exon	ENSG00000087258.9	*GNAO1*	−5.55	1.09 × 10^−^^20^
chr10:96818092-96827448	exon	ENSG00000138115.9	*CYP2C8*	−3.81	3.32 × 10^−^^19^
chr8:62593527-62596747	exon	ENSG00000198363.11	*ASPH*	2.73	7.18 × 10^−^^17^
chr7:87068983-87069718	exon	ENSG00000005471.11	*ABCB4*	−2.65	2.34 × 10^−^^16^
chr12:56871444-56872046	exon	ENSG00000135423.8	*GLS2*	−5.25	3.61 × 10^−^^16^
chr5:113740135-113740553	exon	ENSG00000080709.10	*KCNN2*	−4.42	4.26 × 10^−^^16^
chr6:161157919-161162449	exon	ENSG00000122194.14	*PLG*	−2.94	6.63 × 10^−^^16^
chr4:128995615-128999117	exon	ENSG00000138709.13	*LARP1B*	−1.74	2.69 × 10^−^^15^
chr1:225140372-225156576	exon	ENSG00000185842.10	*DNAH14*	5.36	4.75 × 10^−^^15^
chr16:87935518-87936126	exon	ENSG00000174990.3	*CA5A*	−3.30	5.00 × 10^−^^15^
chr1:225140372-225161855	exon	ENSG00000185842.10	*DNAH14*	2.67	2.57 × 10^−^^14^
chr7:87031149-87032597	exon	ENSG00000005471.11	*ABCB4*	−4.37	2.99 × 10^−^^14^
chr11:122162992-122165713	intron	ENSG00000255090.1	*RP11-820L6.1*	−4.48	5.70 × 10^−^^14^
chr4:1902353-1936989	exon	ENSG00000109685.13	*WHSC1*	3.52	7.04 × 10^−^^14^
chr13:46577274-46619651	exon	ENSG00000123200.12	*ZC3H13*	−2.56	5.98 × 10^−^^13^
chr8:131370263-131374017	exon	ENSG00000153317.10	*ASAP1*	2.11	9.06 × 10^−^^13^

p_adj: adjusted *p*-value after multiple test correction.

**Table 3:** Summary of differentially expressed circRNAs associated with differentially methylated sites in HCC.

Dysregulated circRNAs	Number (by Region)		Hypomethylated Sites	Hypermethylated Sites
Upregulated	195	31 (After2000)	5	23
		155 (Interior)	17	179
		32 (Pre2000)	3	27
Downregulated	134	23 (After2000)	8	19
		102 (Interior)	30	113
		19 (Pre2000)	3	20

**Table 4:** Summary of parental genes of differentially expressed circRNAs with or without differentially methylated sites.

Genes	Expression	Number	Methylation	Promoter	Gene body	Number
Parental genes of upregulated circRNAs	With DE	436	With DM	121	345	367
			Without DM	237	88	69
			Without probe	NA	NA	NA
	Without DE	187	With DM	54	125	146
			Without DM	86	59	38
			Without probe	NA	NA	3
Parental genes of downregulated circRNAs	With DE	337	With DM	84	266	283
			Without DM	180	66	49
			Without probe	NA	NA	NA
	Without DE	147	With DM	32	107	112
			Without DM	84	32	29
			Without probe	NA	NA	6

DE: differentially expressed. DM: differentially methylated.

## Data Availability

All data that support the findings of this study are included in the article.

## Supplementary Materials

The following are available online at https://www.mdpi.com/article/10.3390/biomedicines9060657/s1, Figure S1. Gene Ontology (GO) and KEGG pathway enrichment analysis of the parental genes of differentially expressed circRNAs in HCC. Figure S2. Gene Ontology (GO) and KEGG pathway enrichment analysis of the parental genes with no significant expression change corresponding to the differentially expressed circRNAs. Figure S3. Volcano plot shows differentially expressed circRNAs with differentially methylated sites in HCC. Figure S4. Normalized distribution of differentially expressed circRNAs with (blue) or without (yellow) differentially methylated sites across human chromosomes. Figure S5 Target genes correlated to survival curves with significance in upregulated network. Table S1 Summary of bioinformatics tools used in this study. Table S2 Characteristics of DE circRNAs associated with DNA methylation.
