# Supplementary figures and images for: An Integrative Transcriptomic and Methylation Approach for Identifying Differentially Expressed Circular RNAs Associated with DNA Methylation Change
Source: Biomedicines. 2021 Jun 8;9(6):657. doi: 10.3390/biomedicines9060657 (PMC8227141; doi:10.3390/biomedicines9060657)

Supplemental Figure S1

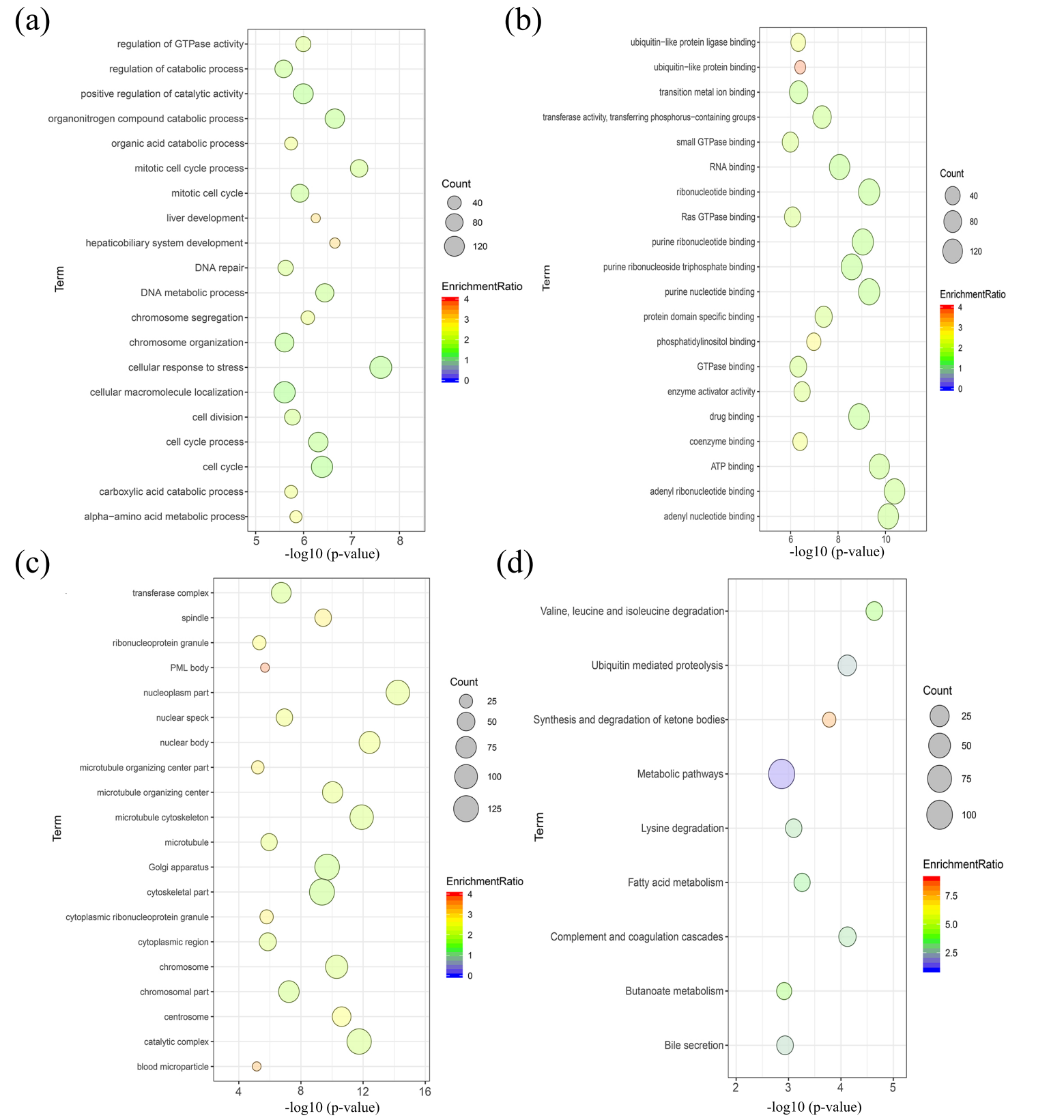

Supplement: Supplementary file 1 [file biomedicines-09-00657-s001.zip › biomedicines-1212508 supp/Supplementary file/Figure-S1_SuppInfo.pdf]

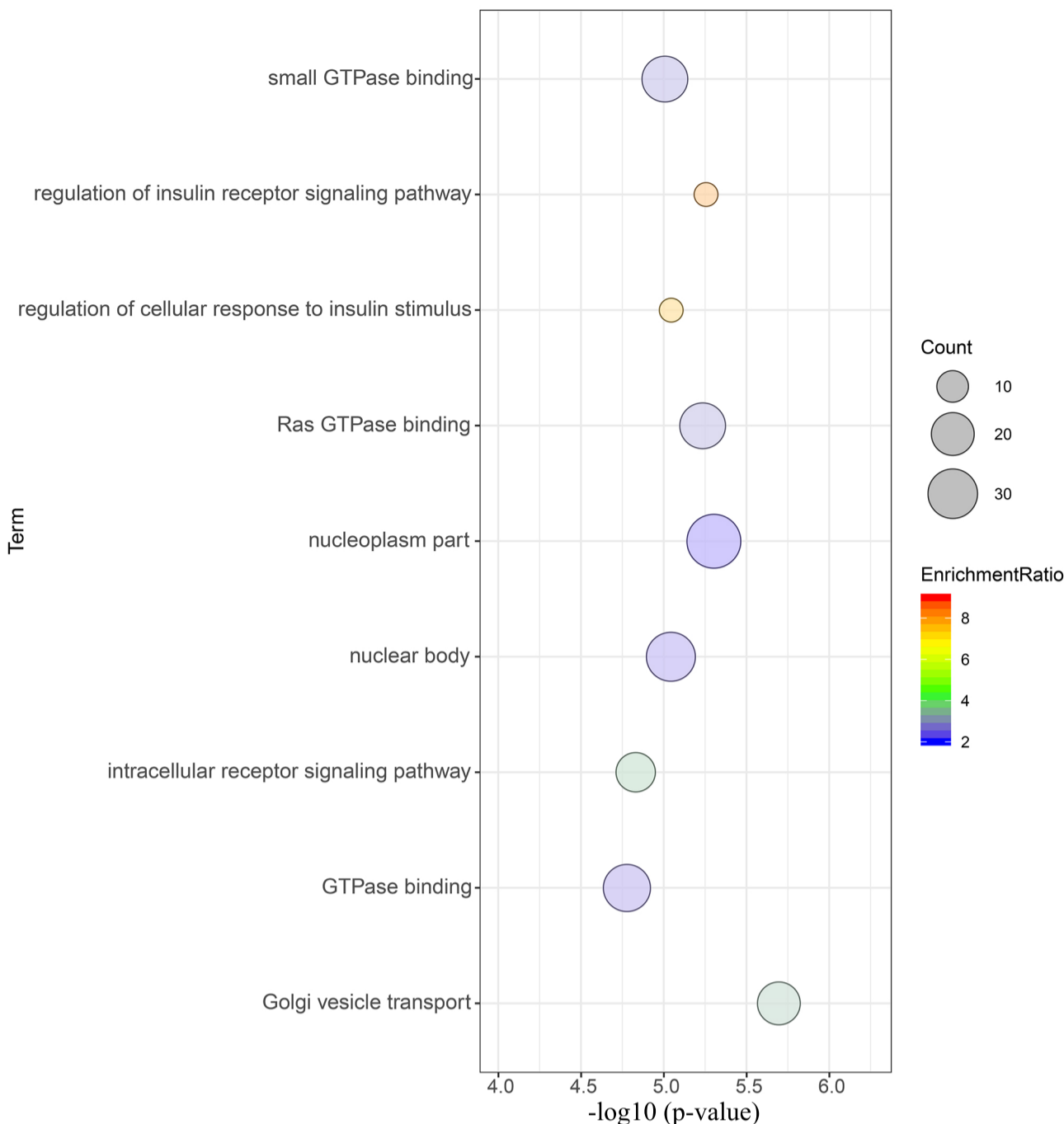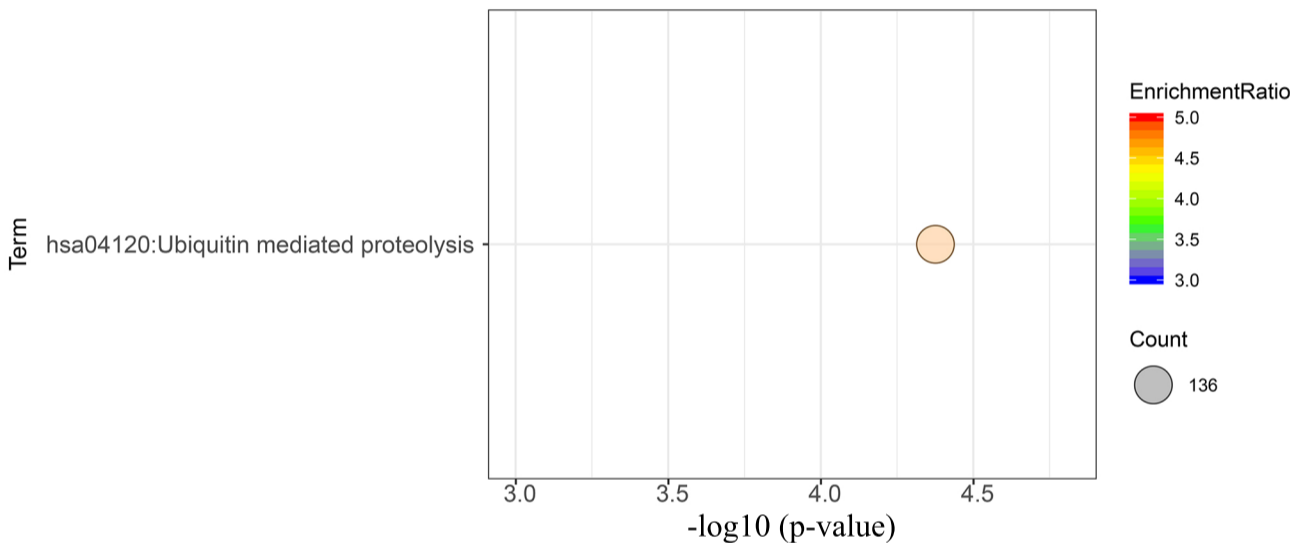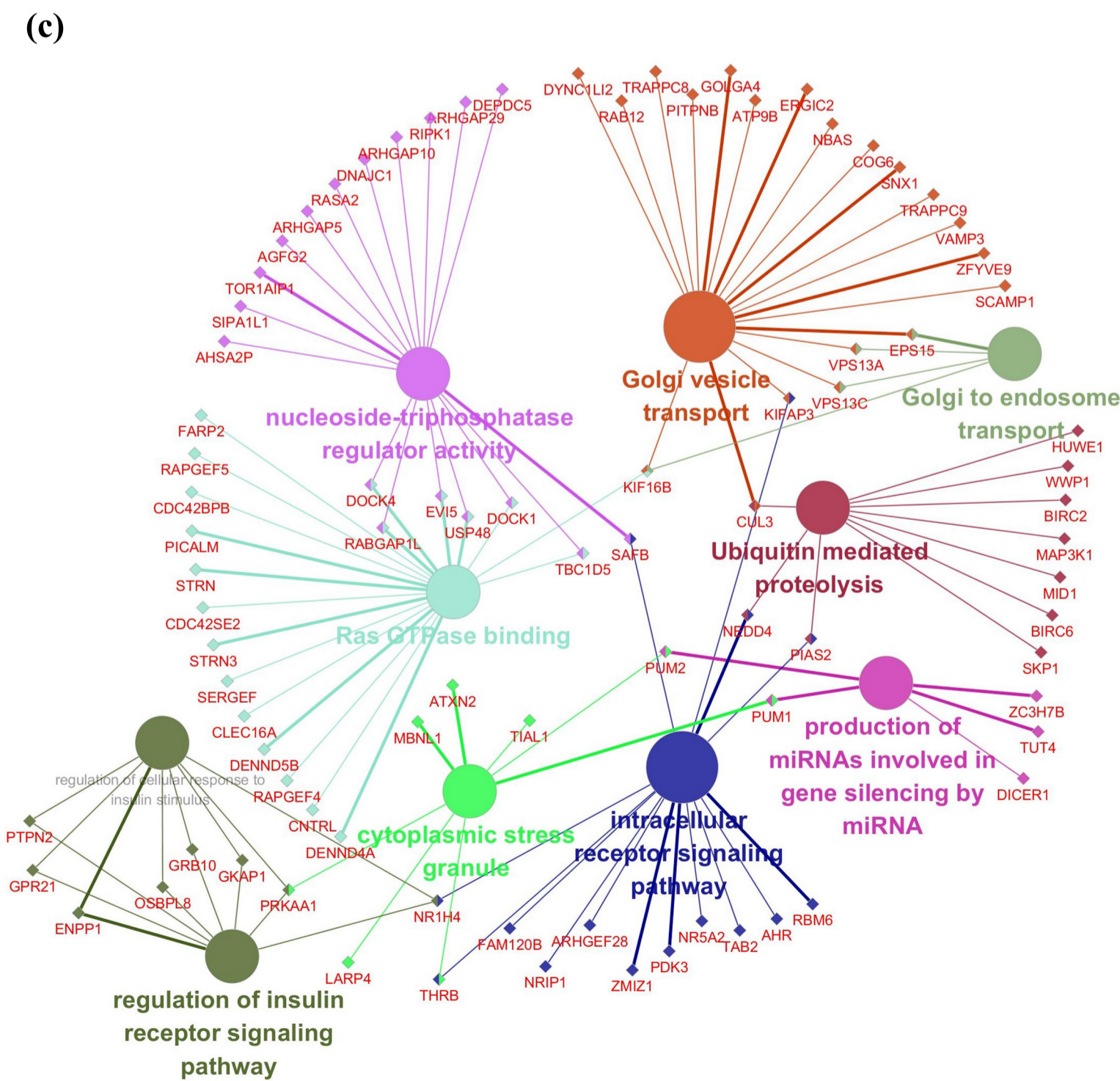

Supplement: Supplementary file 1 [file biomedicines-09-00657-s001.zip › biomedicines-1212508 supp/Supplementary file/Figure-S2_SuppInfo.pdf]

Supplemental Figure S3

Tumor vs. Normal

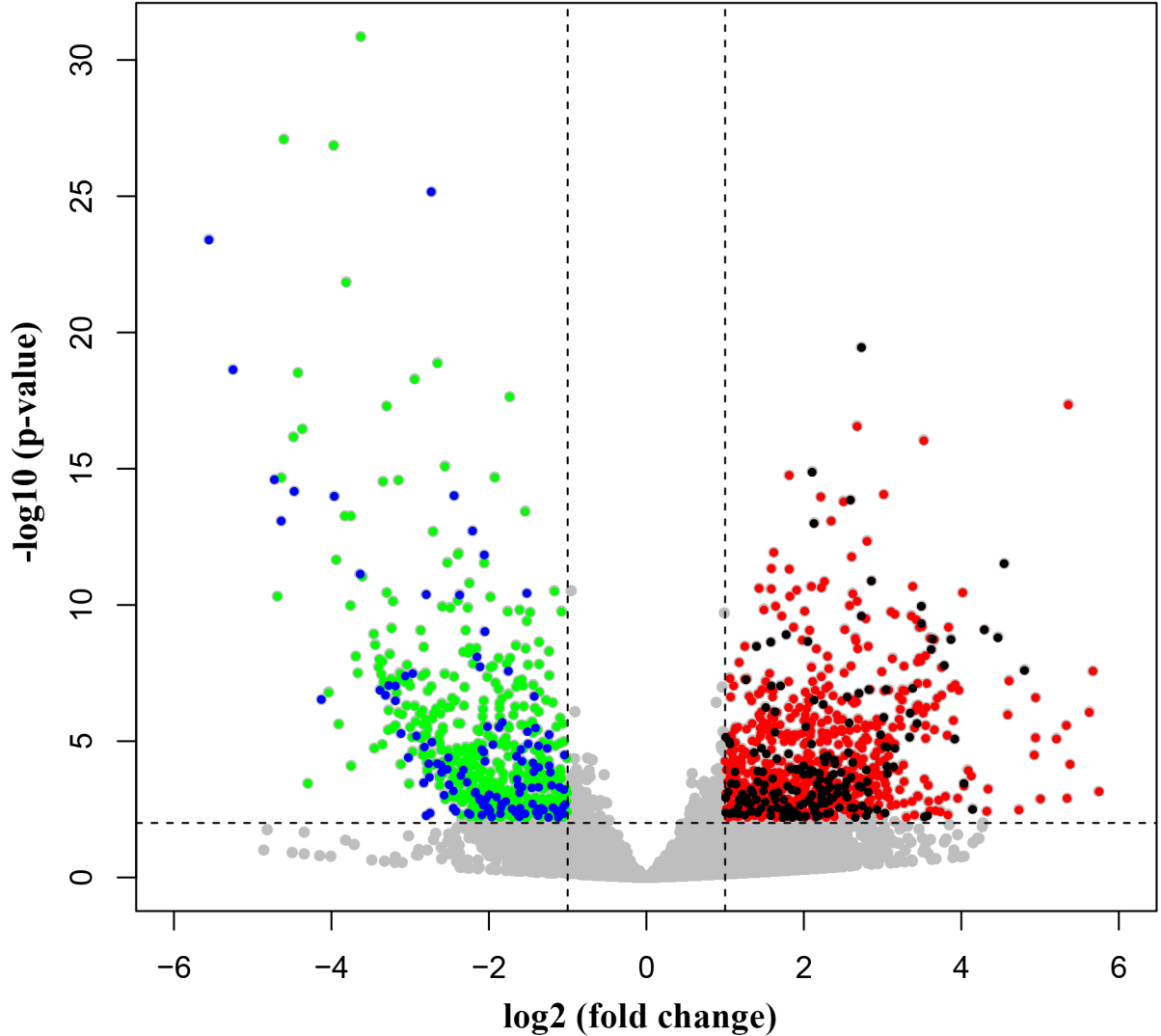

Supplement: Supplementary file 1 [file biomedicines-09-00657-s001.zip › biomedicines-1212508 supp/Supplementary file/Figure-S3_SuppInfo.pdf]

Supplemental Figure S4

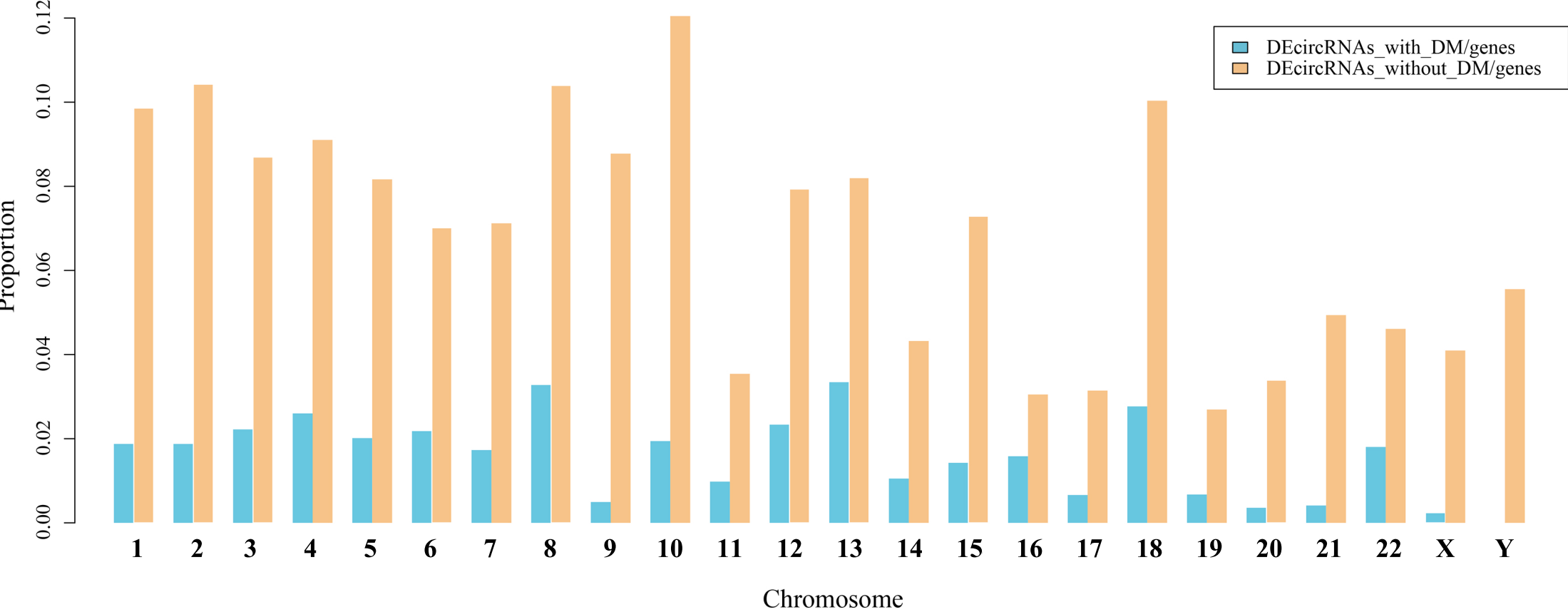

Supplement: Supplementary file 1 [file biomedicines-09-00657-s001.zip › biomedicines-1212508 supp/Supplementary file/Figure-S4_SuppInfo.pdf]

# Supplemental Figure S5

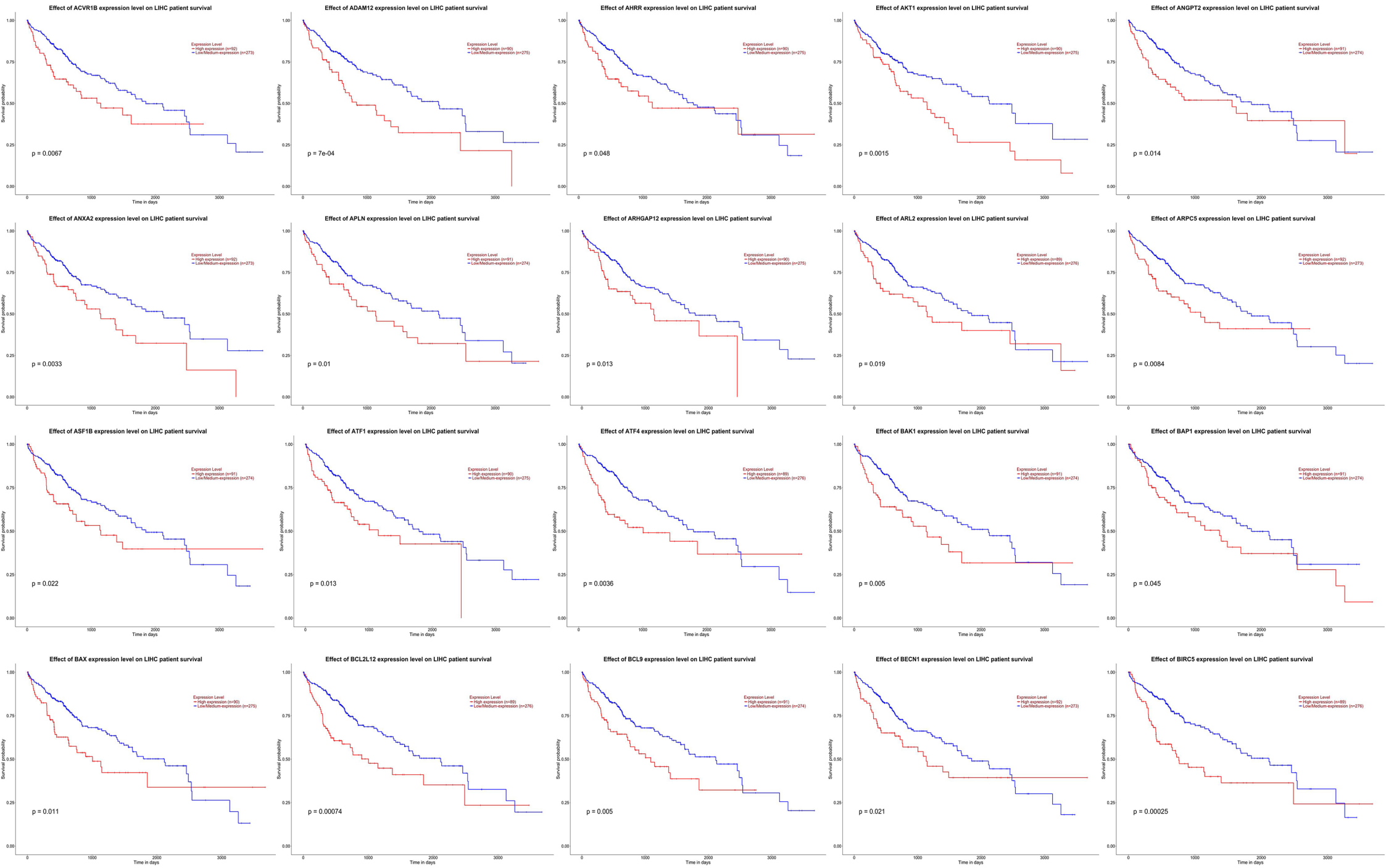

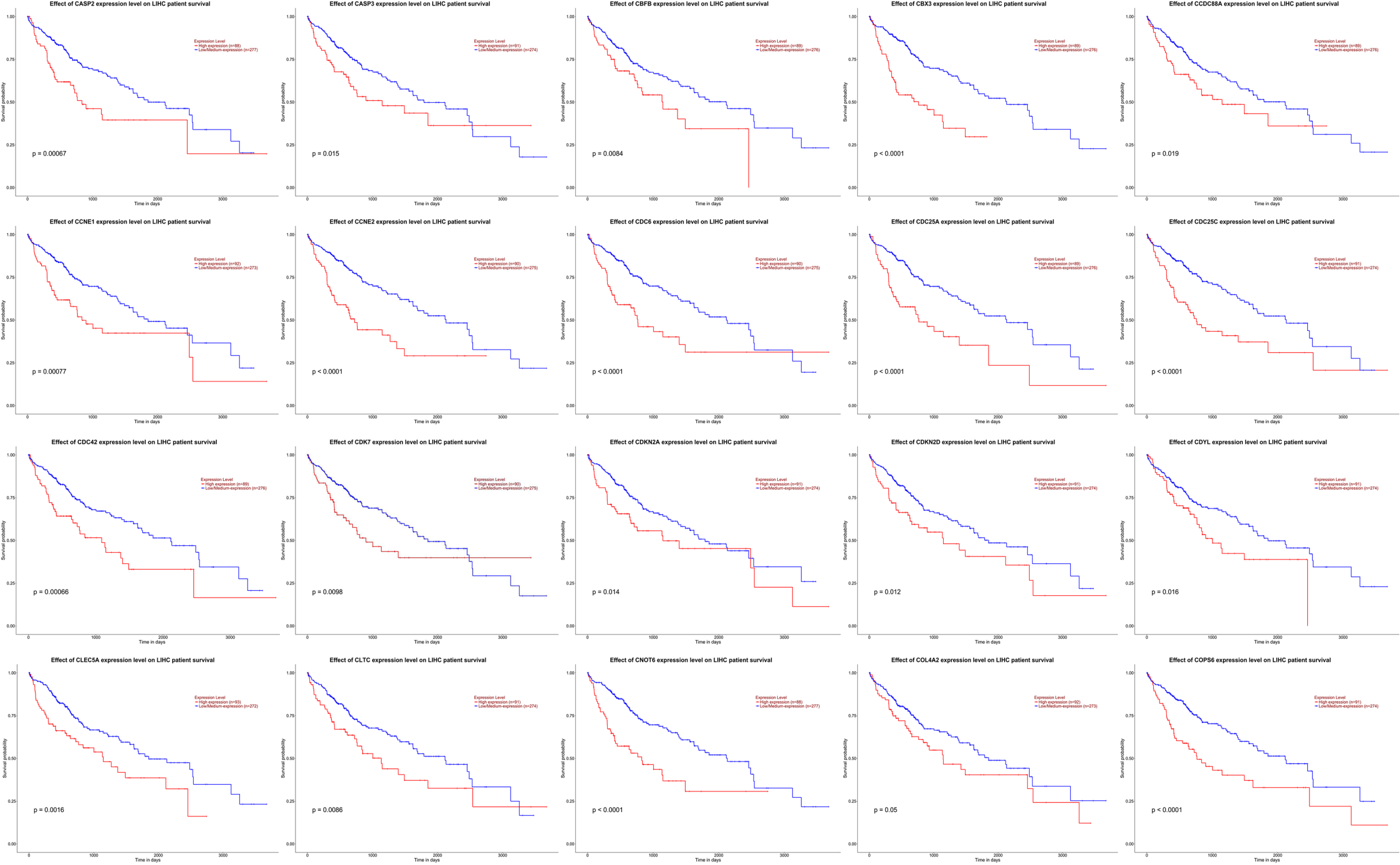

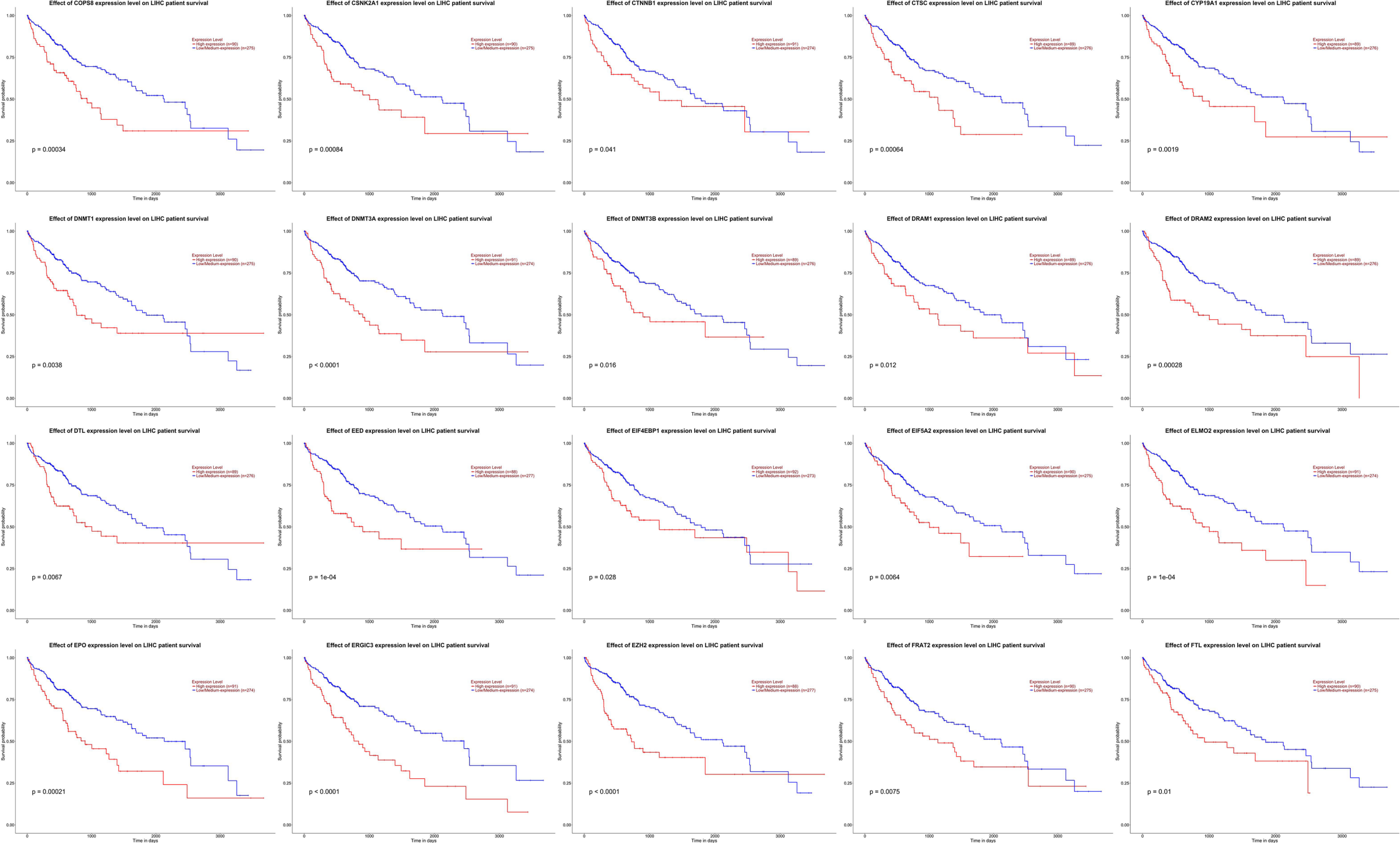

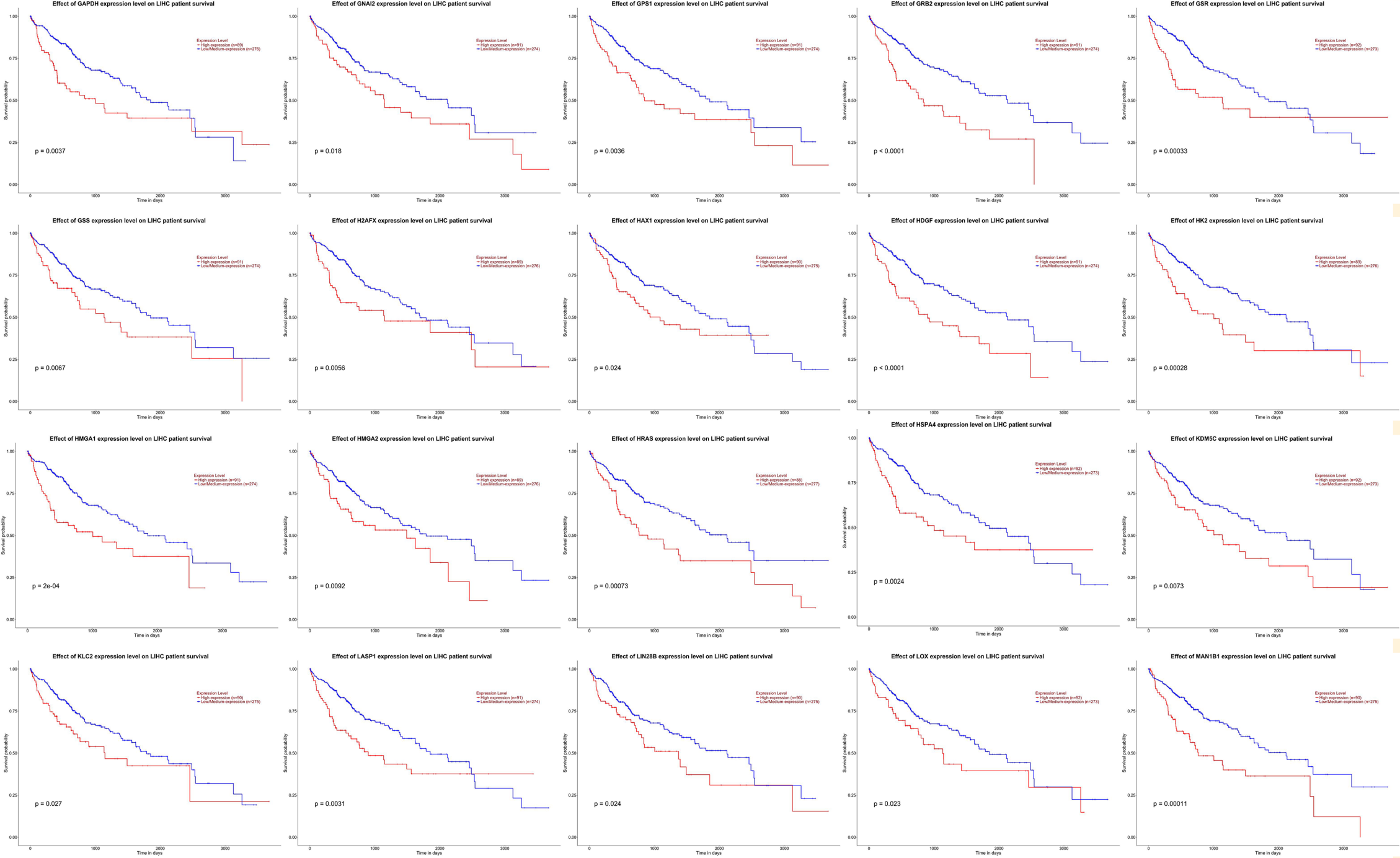

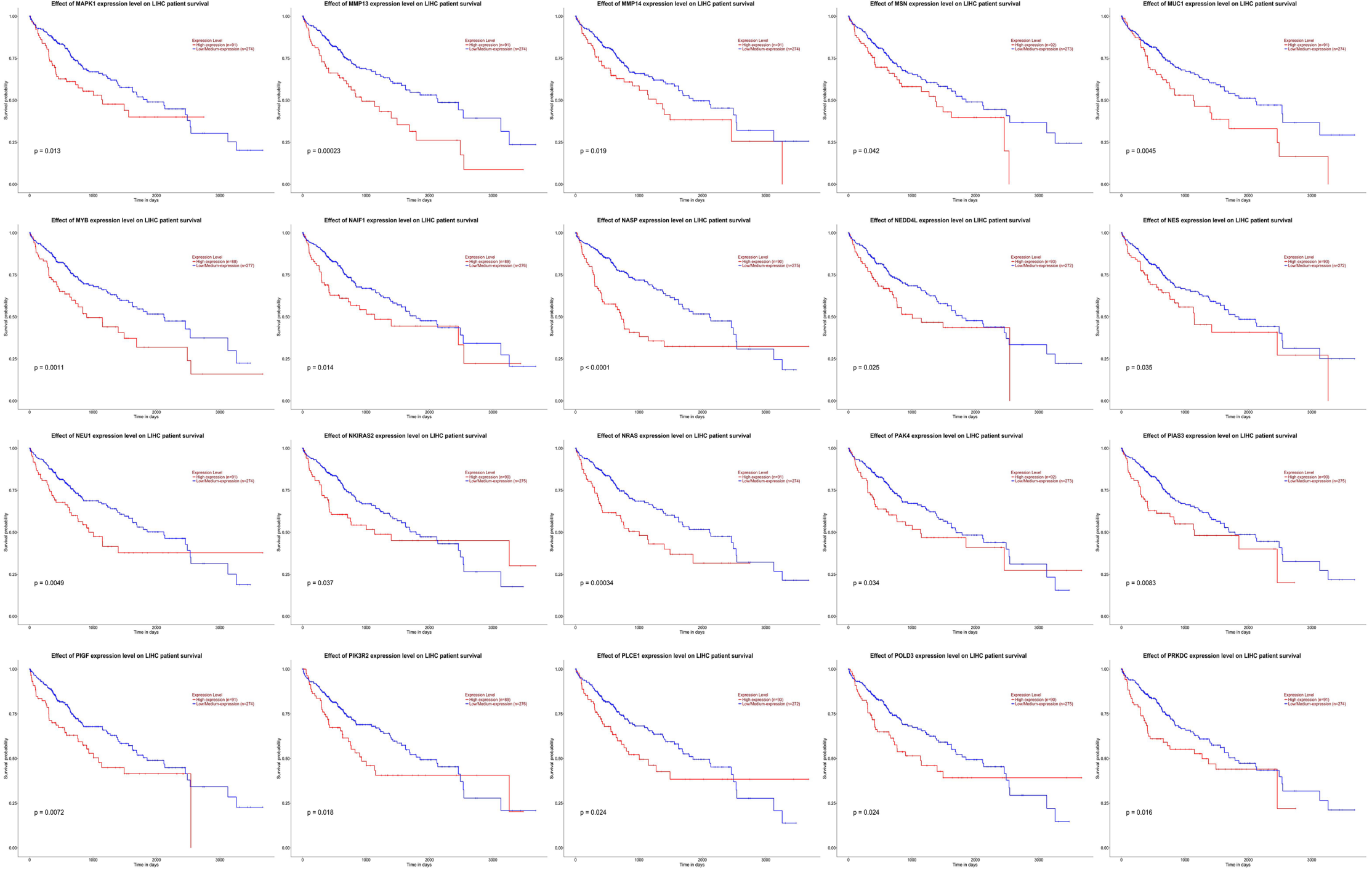

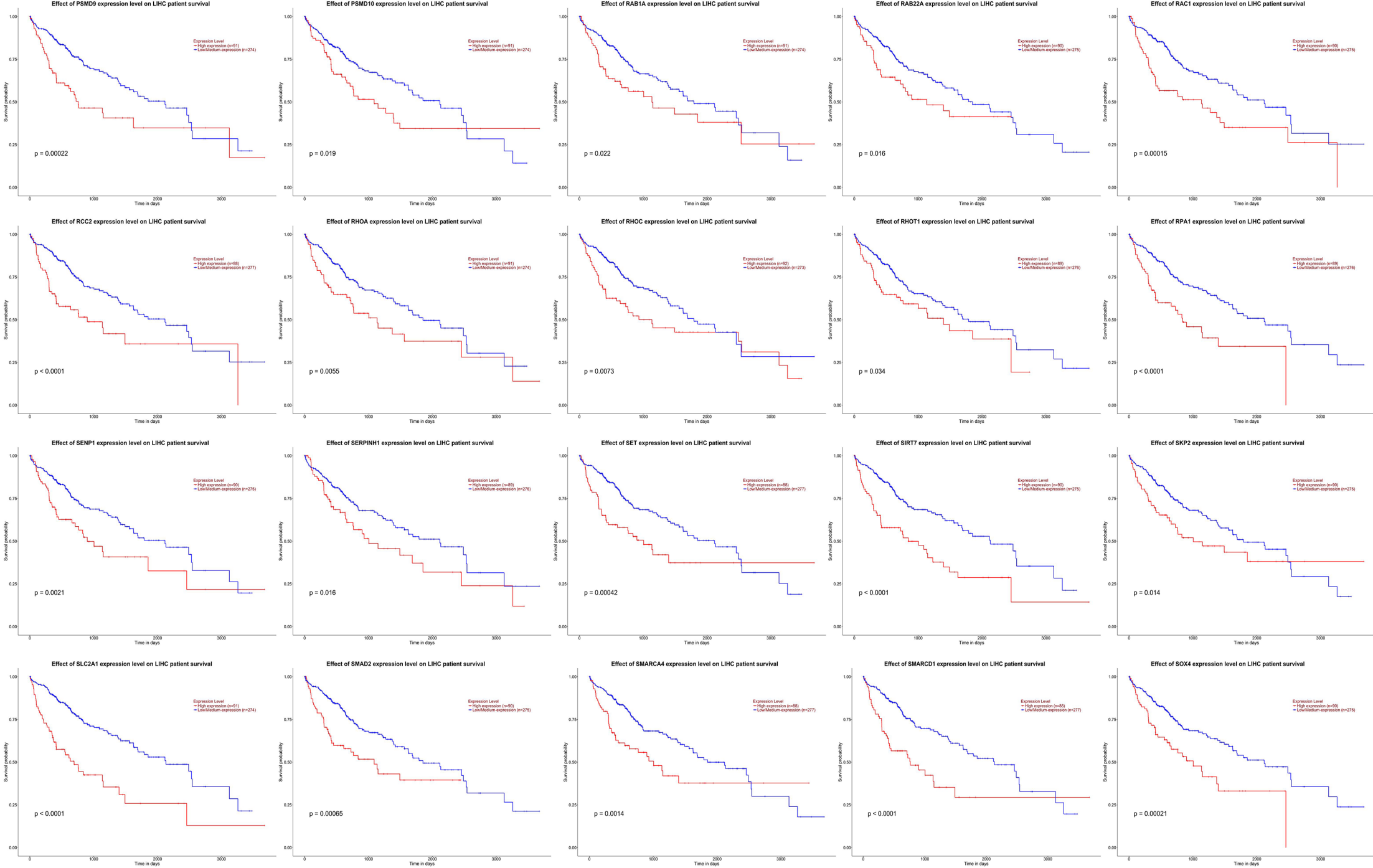

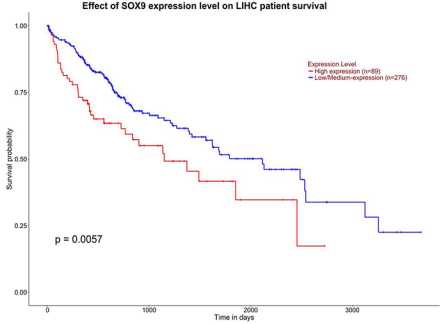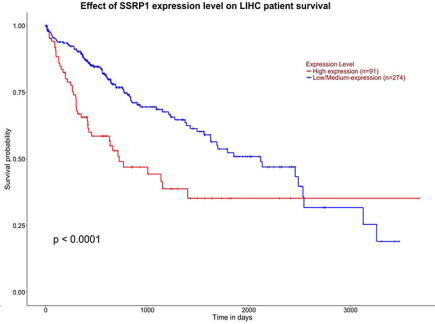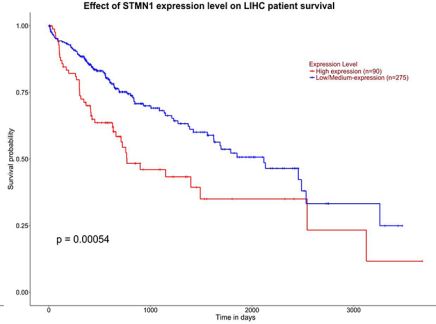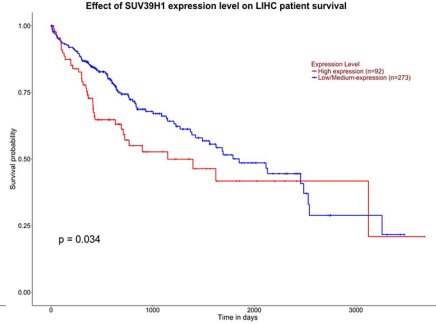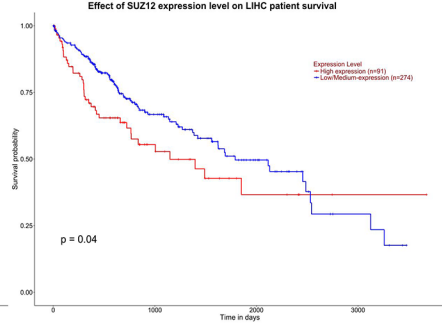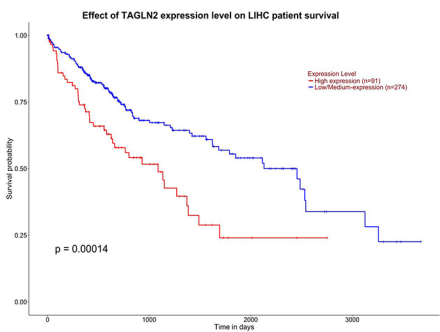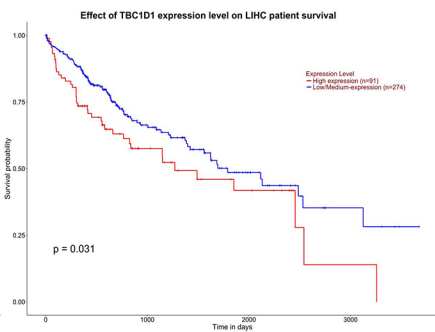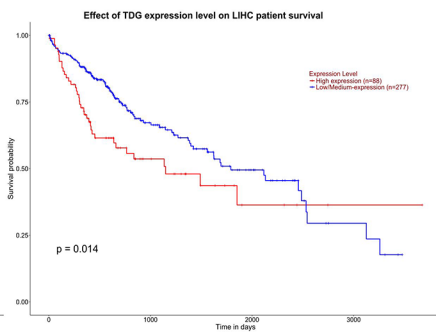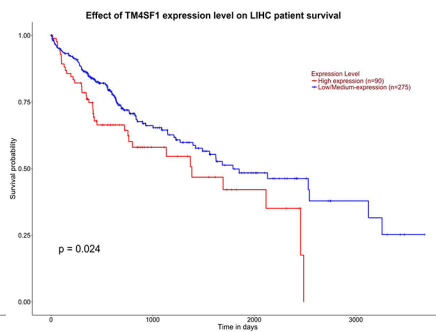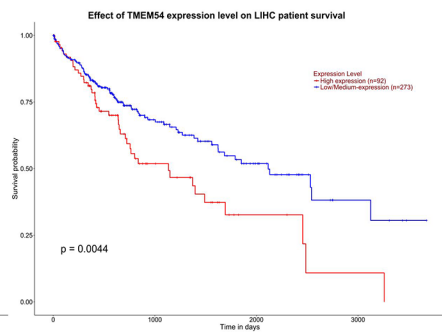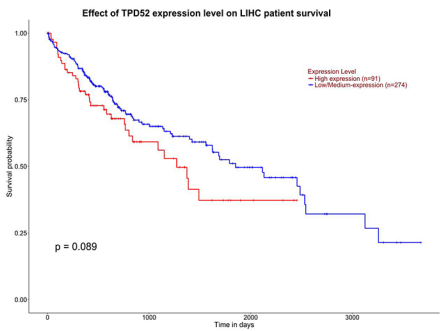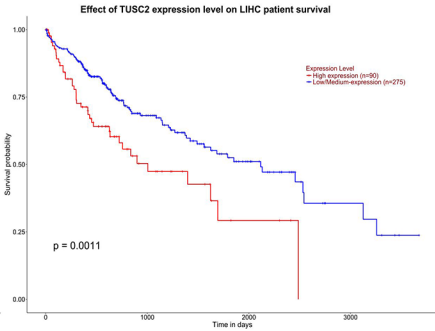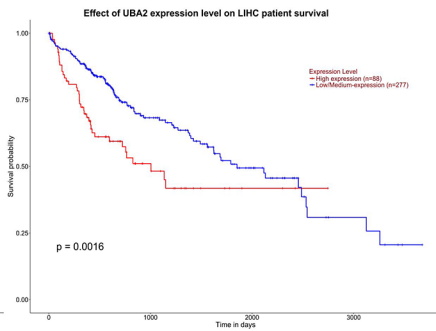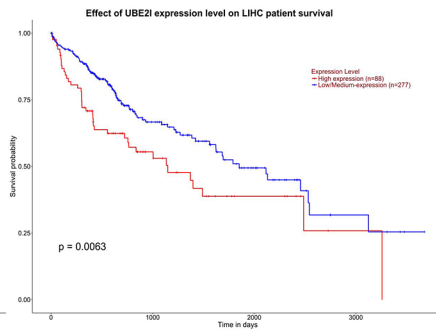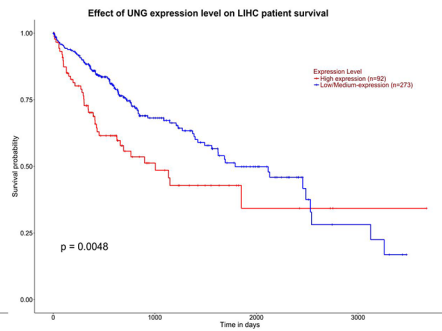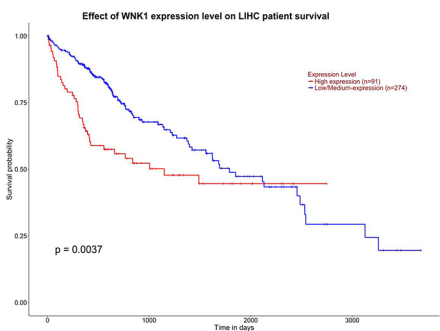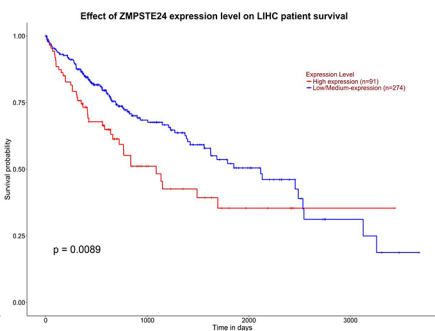

Supplement: Supplementary file 1 [file biomedicines-09-00657-s001.zip › biomedicines-1212508 supp/Supplementary file/Figure-S5_SuppInfo.pdf]
